# Author Correction: The SEQC2 epigenomics quality control (EpiQC) study

**DOI:** 10.1186/s13059-021-02573-y

**Published:** 2021-12-23

**Authors:** Jonathan Foox, Jessica Nordlund, Claudia Lalancette, Ting Gong, Michelle Lacey, Samantha Lent, Bradley W. Langhorst, V. K. Chaithanya Ponnaluri, Louise Williams, Karthik Ramaswamy Padmanabhan, Raymond Cavalcante, Anders Lundmark, Daniel Butler, Christopher Mozsary, Justin Gurvitch, John M. Greally, Masako Suzuki, Mark Menor, Masaki Nasu, Alicia Alonso, Caroline Sheridan, Andreas Scherer, Stephen Bruinsma, Gosia Golda, Agata Muszynska, Paweł P. Łabaj, Matthew A. Campbell, Frank Wos, Amanda Raine, Ulrika Liljedahl, Tomas Axelsson, Charles Wang, Zhong Chen, Zhaowei Yang, Jing Li, Xiaopeng Yang, Hongwei Wang, Ari Melnick, Shang Guo, Alexander Blume, Vedran Franke, Inmaculada Ibanez de Caceres, Carlos Rodriguez-Antolin, Rocio Rosas, Justin Wade Davis, Jennifer Ishii, Dalila B. Megherbi, Wenming Xiao, Will Liao, Joshua Xu, Huixiao Hong, Baitang Ning, Weida Tong, Altuna Akalin, Yunliang Wang, Youping Deng, Christopher E. Mason

**Affiliations:** 1 Department of Physiology and Biophysics, Weill Cornell Medicine, New York, NY USA; 2 The HRH Prince Alwaleed Bin Talal Bin Abdulaziz Alsaud Institute for Computational Biomedicine, Weill Cornell Medicine, New York, NY USA; 3 Department of Medical Sciences and Science for Life Laboratory, Uppsala University, Uppsala, Sweden; 4 EATRIS ERIC- European Infrastructure for Translational Medicine, De Boelelaan 1118, 1081 HZ Amsterdam, The Netherlands; 5 BRCF Epigenomics Core, University of Michigan Medicine, Ann Arbor, MI 48109 USA; 6 Department of Quantitative Health Sciences, University of Hawaii John A. Burns School of Medicine, Honolulu, HI 96813 USA; 7 Tulane University, New Orleans, LA 70118 USA; 8 AbbVie Genomics Research Center, 1 N. Waukegan Rd, North Chicago, IL 60036 USA; 9 New England Biolabs, Ipswich, MA 01938 USA; 10 Albert Einstein College of Medicine, Bronx, NY 10461 USA; 11 Division of Hematology/Oncology, Department of Medicine, Epigenomics Core Facility, Weill Cornell Medicine, New York, NY USA; 12 Institute for Molecular Medicine Finland (FIMM), University of Helsinki, Helsinki, Finland; 13 Illumina, Inc, Madison, WI 53705 USA; 14 Faculty of Biochemistry, Biophysics and Biotechnology, Jagiellonian University, Krakow, Poland; 15 Małopolska Centre of Biotechnology, Jagiellonian University, Krakow, Poland; 16 New York Genome Center, New York, NY 10013 USA; 17 Center for Genomics, School of Medicine, Loma Linda University, Loma Linda, CA 92350 USA; 18 Department of Allergy and Clinical Immunology, State Key Laboratory of Respiratory Disease, Guangzhou Institute of Respiratory Health, the First Affiliated Hospital of Guangzhou Medical University, Guangzhou, Guangdong China; 19 Department of Neurology, The Second Affiliated Hospital of Zhengzhou University, Zhengzhou, 450014 China; 20 Development of Medicine, the University of Chicago, Chicago, IL 60637 USA; 21 Department of Neurology, the Second Affiliated Hospital of Zhengzhou University, Zhengzhou, 450014 China; 22 Bioinformatics and Omics Data Science Platform, Berlin Institute for Medical Systems Biology, Max Delbrueck Center for Molecular Medicine, Berlin, Germany; 23 Cancer Epigenetics Laboratory, INGEMM, IdiPAZ, Madrid, Spain; 24 CMINDS Research Center, Francis College of Engineering, University of Massachusetts Lowell, Lowell, MA 01854 USA; 25 Center for Devices and Radiological Health, Food and Drug Administration, 10903 New Hampshire Ave, Silver Spring, MD 20993 USA; 26 Division of Bioinformatics and Biostatistics, National Center for Toxicological Research, Food and Drug Administration, 3900 NCTR Road, Jefferson, AR 72079 USA; 27 The Feil Family Brain and Mind Research Institute, New York, NY USA; 28 The WorldQuant Initiative for Quantitative Prediction, Weill Cornell Medicine, New York, NY USA

**Correction to: Genome Biol 22, 332 (2021)**

**https://doi.org/10.1186/s13059-021-02529-2**

Following publication of the original article [1], the authors identified the following errors:

1. Jonathan Foox, Jessica Nordlund, Claudia Lalancette, Ting Gong, Michelle Lacey and Samantha Lent are co-first authors.
2. Affiliation 21 was incorrectly published. It should be: Department of Neurology, the Second Affiliated Hospital of Zhengzhou University. Zhengzhou, China, 450014.
3. Additional file 1 should contain the supplementary figures. The additional file 1 in this correction article has been updated accordingly.
4. Reference 9 in the original article has been updated to: Vaisvila R, Ponnaluri VKC, Sun Z, Langhorst BW, Saleh L, Guan S, Dai N, Campbell MA, Sexton BS, Marks K, Samaranayake M, Samuelson JC, Church HE, Tamanaha E, Corrêa IR Jr., Pradhan S, Dimalanta ET, Evans TC Jr., Williams L, Davis TB. Enzymatic methyl sequencing detects DNA methylation at single-base resolution from picograms of DNA. Genome Res. 2021 Jun 17;31(7):1280–9. doi: 10.1101/g.266551.120. Epub ahead of print. PMID: 34140313; PMCID: PMC8256858.

The original article [1] has been corrected.

## Supplementary Information

**Additional file 1.** Contains the supplementary figures (Supplementary Figure 1–14).
