# Author Correction: Benchmarking transposable element annotation methods for creation of a streamlined, comprehensive pipeline

**DOI:** 10.1186/s13059-022-02645-7

**Published:** 2022-03-08

**Authors:** Shujun Ou, Weijia Su, Yi Liao, Kapeel Chougule, Jireh R. A. Agda, Adam J. Hellinga, Carlos Santiago Blanco Lugo, Tyler A. Elliott, Doreen Ware, Thomas Peterson, Ning Jiang, Candice N. Hirsch, Matthew B. Hufford

**Affiliations:** 1 Department of Ecology, Evolution, and Organismal Biology, Iowa State University, Ames, IA 50011 USA; 2 Department of Genetics, Development, and Cell Biology, Iowa State University, Ames, IA 50011 USA; 3 Department of Ecology and Evolutionary Biology, University of California, Irvine, CA 92697 USA; 4 Cold Spring Harbor Laboratory, Harbor, Cold Spring, NY 11724 USA; 5 Centre for Biodiversity Genomics, University of Guelph, Guelph, Ontario N1G 2W1 Canada; 6 USDA-ARS NEA Robert W. Holley Center for Agriculture and Health, Cornell University, Ithaca, NY 14853 USA; 7 Department of Horticulture, Michigan State University, East Lansing, MI 48824 USA; 8 Department of Agronomy and Plant Genetics, University of Minnesota, Saint Paul, MN 55108 USA

**Correction to: Genome Biol 20, 275 (2019)**

**https://doi.org/10.1186/s13059-019-1905-y**

Following publication of the original article [1], the authors identified an error in the author name of Weijia Su.

The incorrect author name is: Weija Su

The correct author name is: Weijia Su

The author group has been updated in this correction article.
