# Supplementary material for: The SEQC2 epigenomics quality control (EpiQC) study
Source: Genome Biol. 2021 Dec 6;22:332. doi: 10.1186/s13059-021-02529-2 (PMC8650396; doi:10.1186/s13059-021-02529-2)
Supplement: Supplementary file 1 — Additional file 1. contains the supplementary figures (Supplementary Figure 1–14). [file 13059_2021_2529_MOESM1_ESM.pdf]

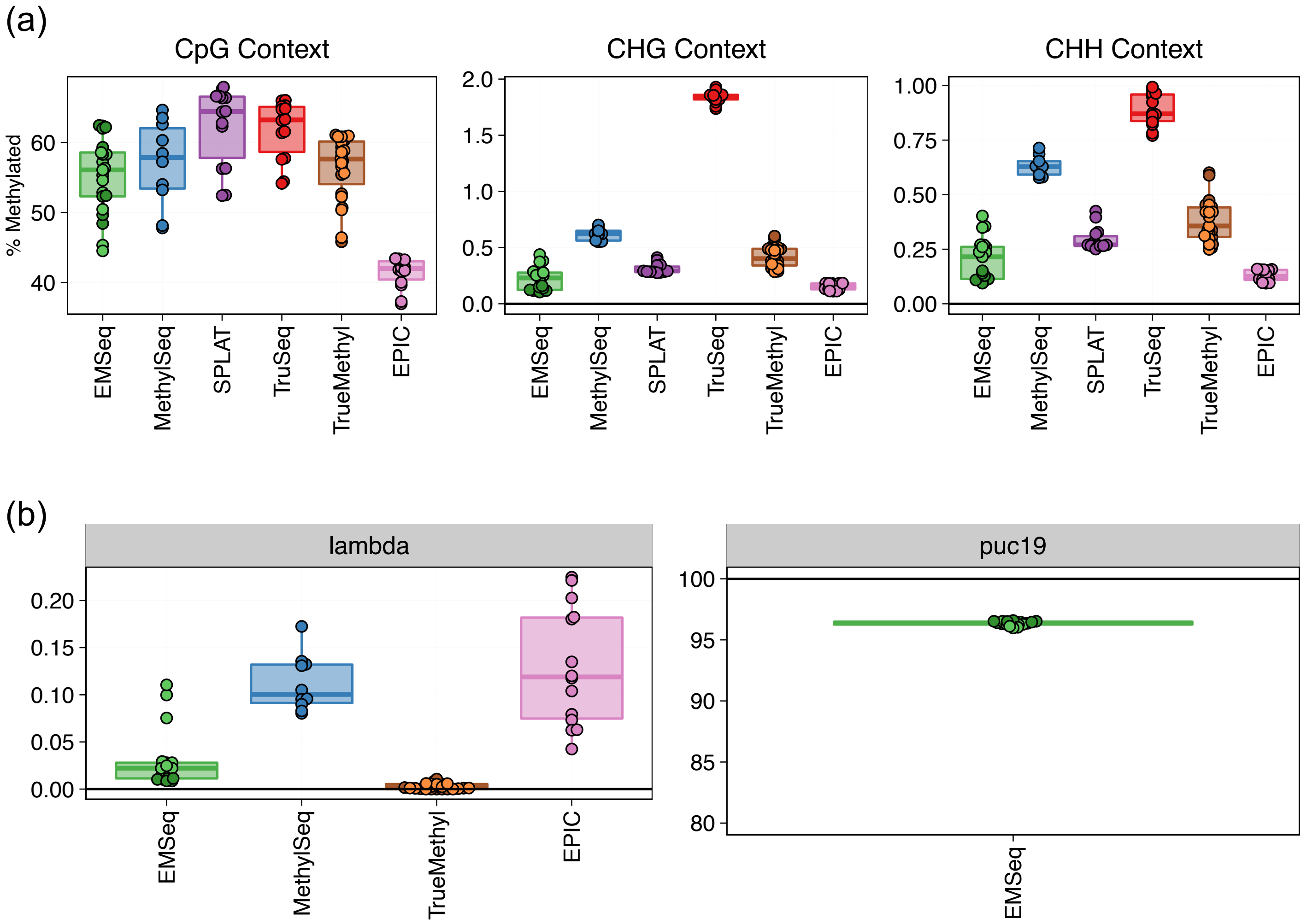

Figure S1: Measurement of sequencing control samples (a) Estimated methylation percentage in CpG, CHG, and CHH contexts per assay. Efficient conversion results in near-zero converted cytosines in CHG and CHH contexts. (b) Estimated methylation percentage in unmethylated controls, showing only assays that had these controls spiked in as a part of their library preparation.

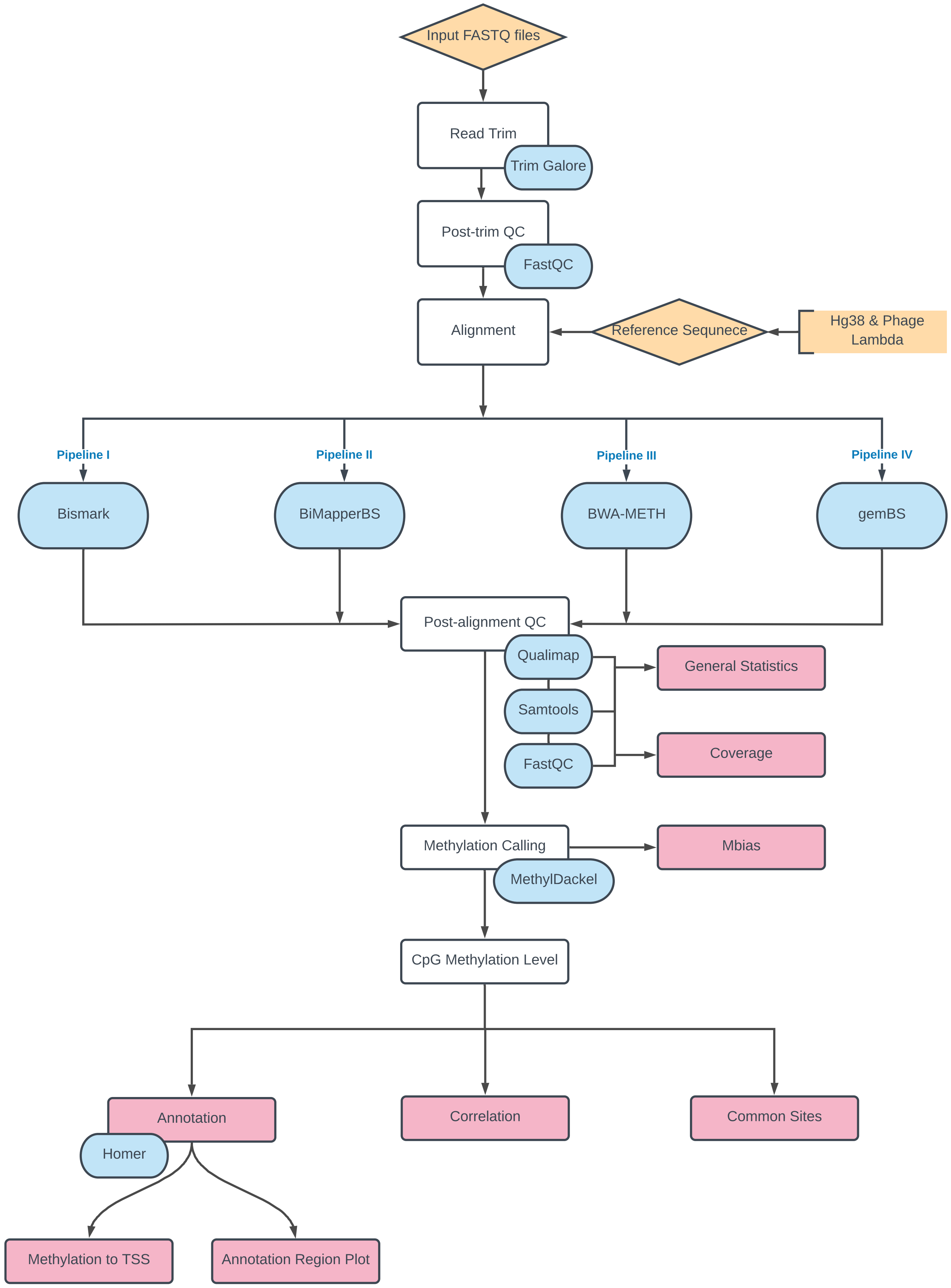

Figure S2: Flowchart showing recommended steps for read quality control, reference-based read alignment, and methylation extraction, for each methylation package analyzed.

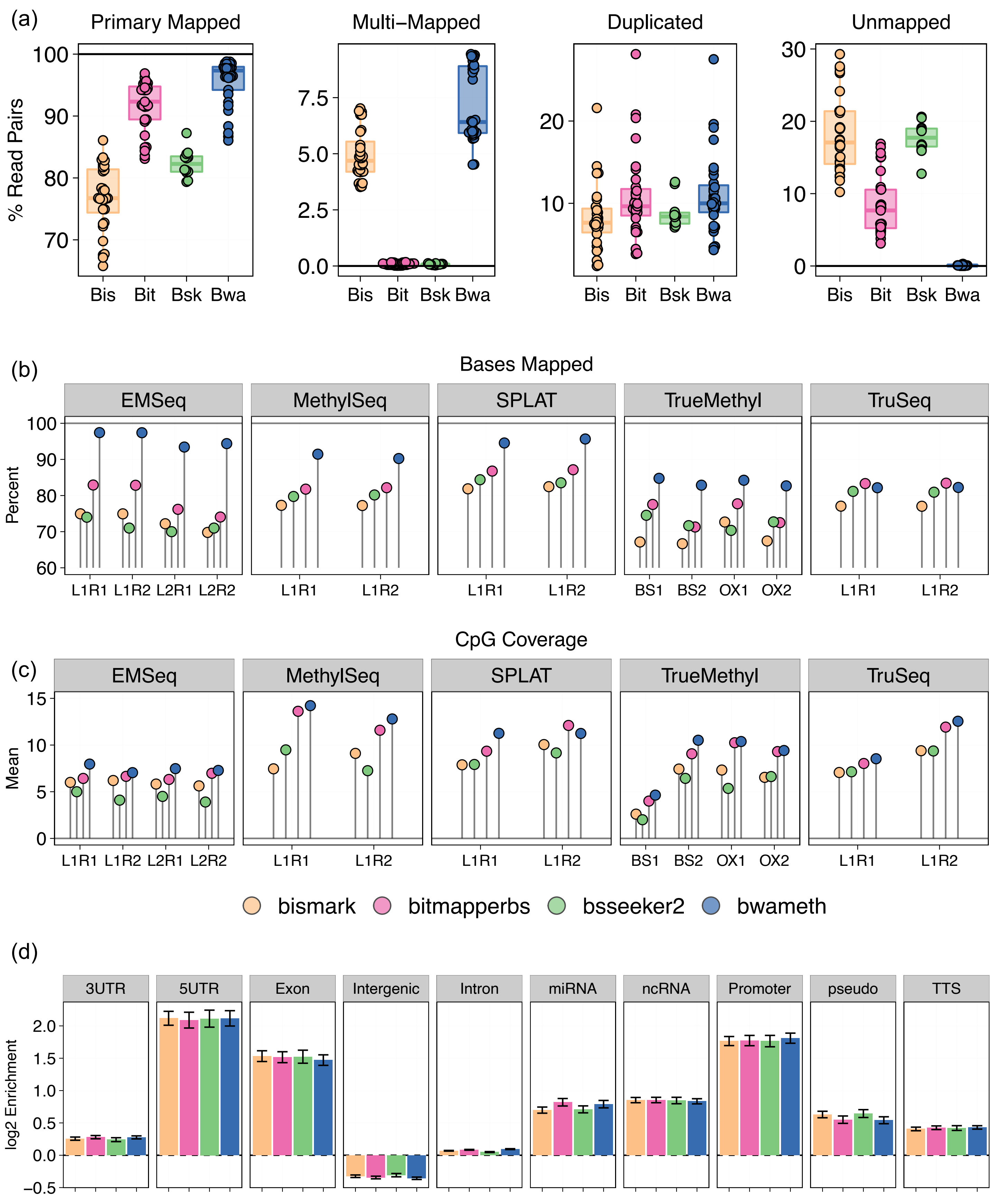

Figure S3: Comparison of outputs for each methylation detection pipeline. All figures show analysis of all HG002 samples for each short read epigenomic assay. (a) Distribution of reference-based read alignment outcomes, including primary mapped reads (both mates mapped in correct orientation within a certain distance), multi-mapped reads (read pairs containing secondary or supplementary alignments), reads marked as PCR or optical duplicates, and unmapped reads. Ambiguous and duplicate reads can be a subset of properly aligned reads. (b) Mapping efficiency per pipeline as measured by the total percentage of reads aligned to the reference genome. L1 and L2 = Lab 1/2; R1 and R2 = Replicate 1/2; BS1 and BS2 = bisulfite treatment replicates 1/2; OX1 and OX2 = oxidative-bisulfite replicates 1/2. (c) The mean coverage per CpG across the genome per pipeline. (d) The regions of the genomes covered per pipeline, measured as log2 enrichment against a null genomic distribution.

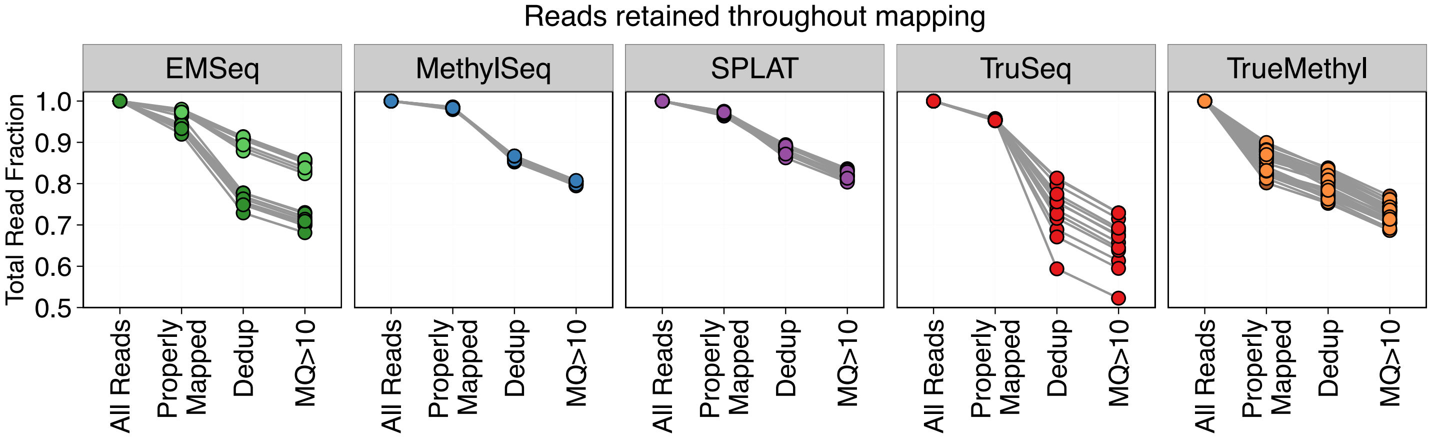

Figure S4: Read retention rate. The fraction of total reads that are retained after each step of the epigenome alignment process is shown per assay. Properly mapped = both mates of a pair were mapped in the correct orientation within a 1kb distance. Dedup = removing reads that are marked as duplicates. MQ = Mapping Quality.

a)

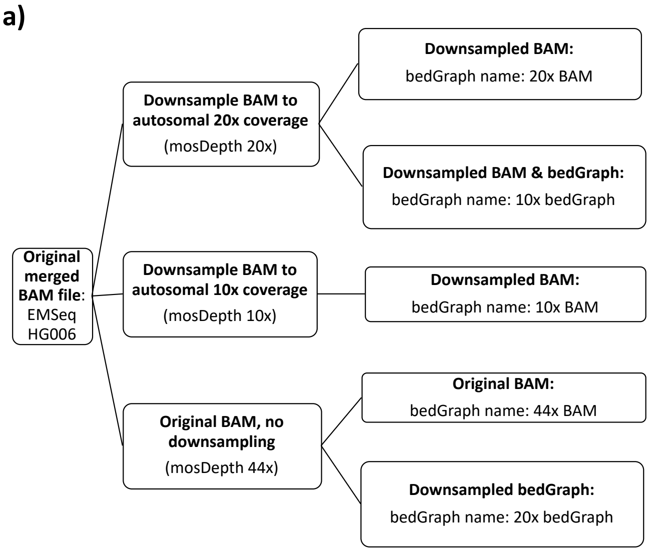

b)

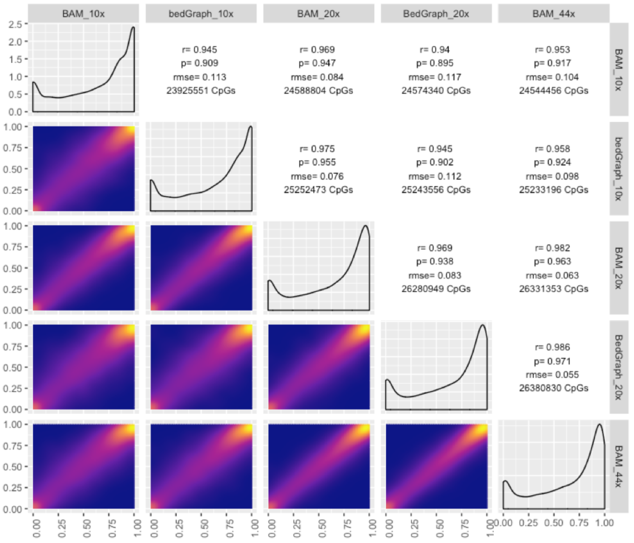

c)

bedGraph name:	10x BAM	10x bedGraph	20x BAM	20x bedGraph	44x BAM
Type of down-sampling	BAM	BAM (20x) -> bedGraph (10x)	BAM	bedGraph	none
Final target coverage	10x	10x	20x	20x	44x
**Beta-value statistics**					
Min.	0.00	0.00	0.00	0.00	0.00
1st Qu.	0.36	0.36	0.35	0.42	0.43
Median	0.77	0.77	0.76	0.79	0.79
Mean	0.64	0.64	0.63	0.66	0.66
3rd Qu.	1.00	1.00	0.94	0.94	0.93
Max.	1.00	1.00	1.00	1.00	1.00
**Coverage statistics**					
Min.	1	1	1	1	1
1st Qu.	7	7	14	16	33
Median	9	10	18	20	39
Mean	9.0	10.0	17.9	20.4	38.4
3rd Qu.	11	12	21	24	44
Max.	1930	1130	1932	2946	5478
99%	18	19	31	35	60
95%	15	16	27	30	53

d)

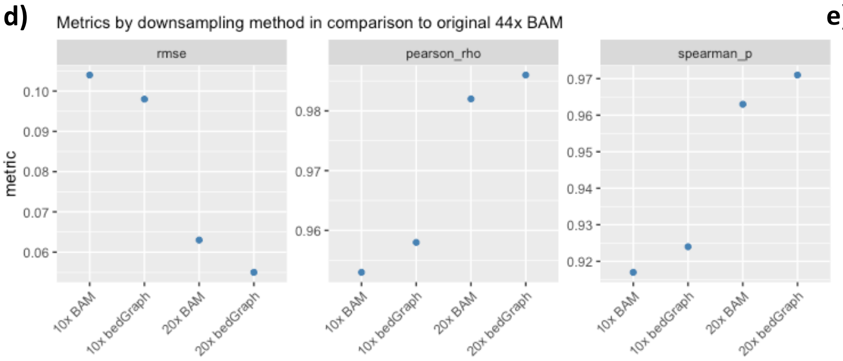

e)

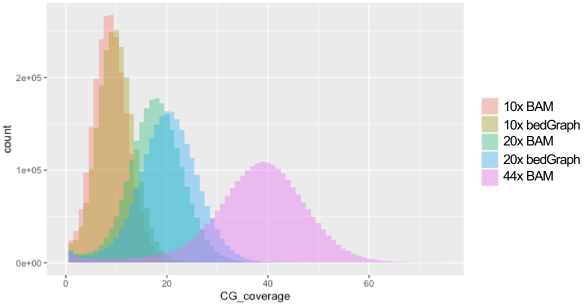

Figure S5: Downsampling evaluation for EMSeq / HG006. (a) Outline of the downsampling procedure and naming scheme of the downsampled libraries. (b) Pairwise correlation matrix of methylation values for the EMSeq HG006 library from Lab 1. Scatter plots of the methylation values are shown in the lower left. Histograms of the methylation values per library are shown across the diagonal. Pairwise Pearson (rho) and Spearman (p) correlation coefficients, root mean square error (RMSE), and the number of CpG dinucleotides with >= 5x coverage in both libraries are shown in the upper right. (c) Statistics over the methylation percentage distributions and observed read coverage of CpG sites in the various bedGraph files. (d) RMSE, Pairwise Pearson (p) and Spearman (rho) correlations between downsampled BAM and bedGraph files in comparison to the original 44x average coverage BAM file. (e) Histograms of the CG dinucelotide read coverage of each bedGraph file prior (44x BAM) to and after downsampling the BAM or bedGraph.

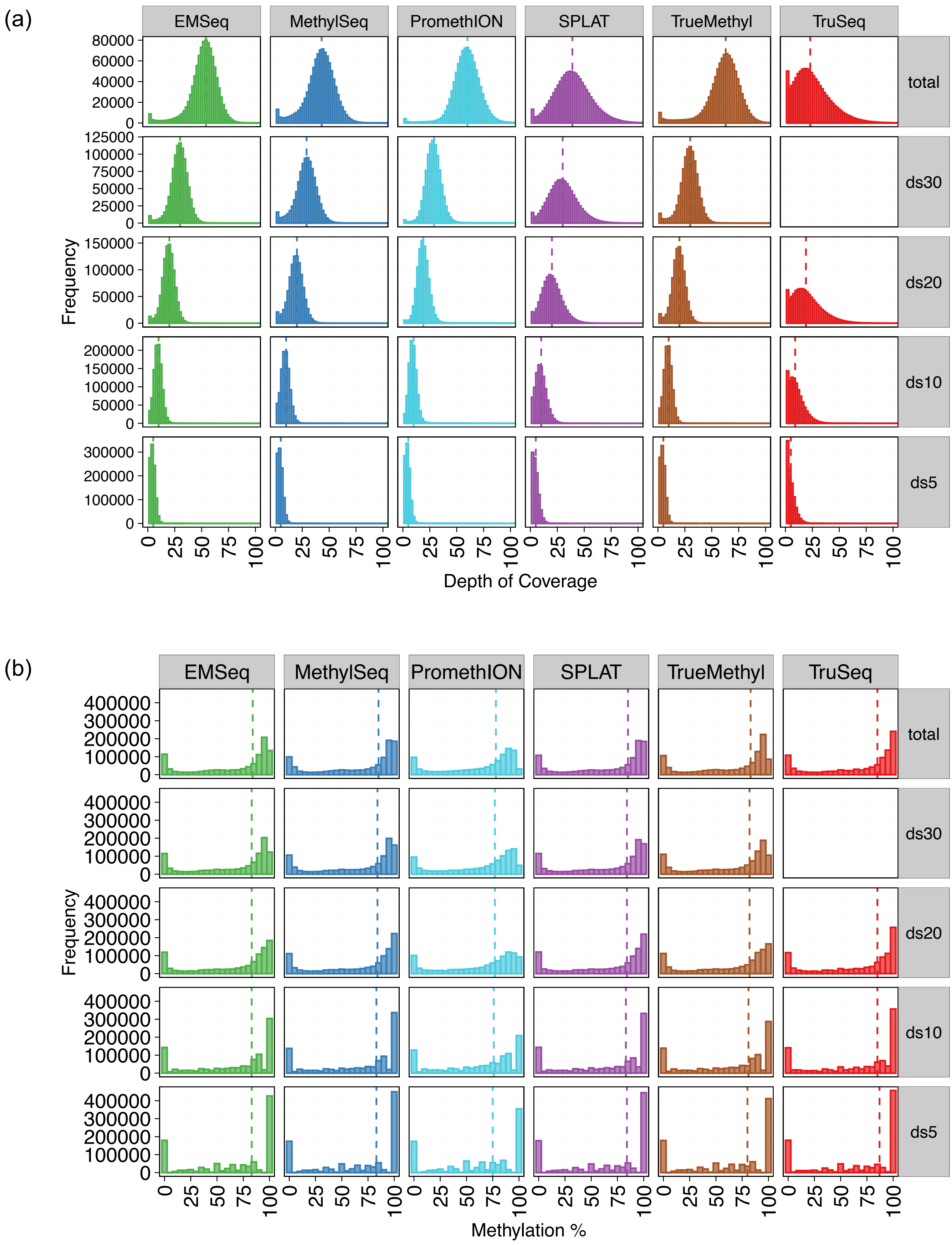

Figure S6: CpG coverage and methylation percentage distributions for complete and downsampled libraries per assay. All values are shown for replicates of HG003. ds = downsample, indicating the mean CpG coverage samples were normalized to. Vertical dotted lines indicate median coverage/methylation percentage. TruSeq could not be downsampled at the 30x level because the total mean depth for TruSeq libraries equaled roughly 30x.

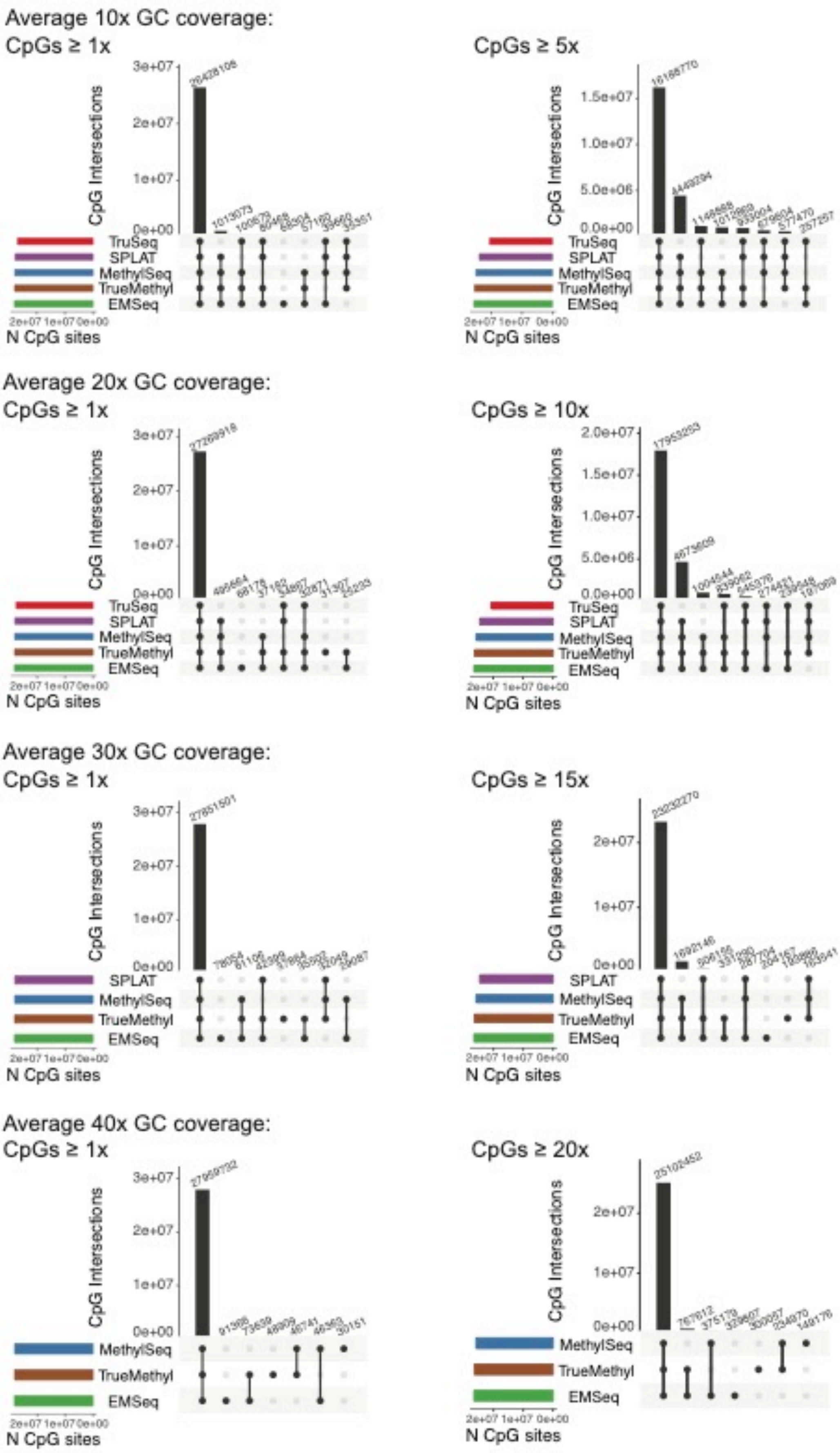

Figure S7: UpSet plots showing shared coverage of CpGs across assays across downsampling schema, with a minimum of 1x cov per CpG on the left and a minimum of 50% of the downsampling scheme on the right (e.g. minimum of 5x coveage for 10x downsampled data).

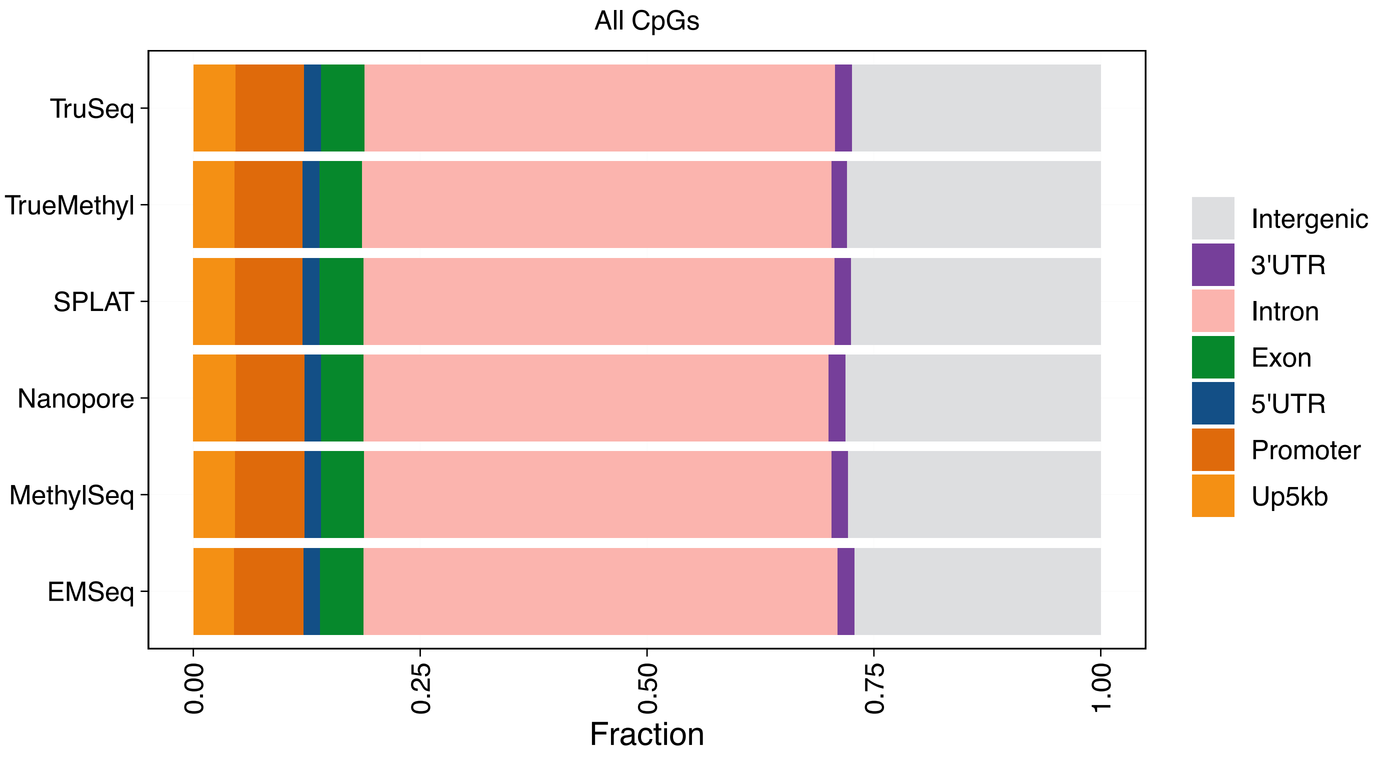

Figure S8: Annotating CpGs covered by each assay using normalized mean 20x coverage data, showing the consistency of coverage genome-wide. Up5kb = 5kb upstream of promoter regions. Promoter = 1kb upstream of transcript start sites.

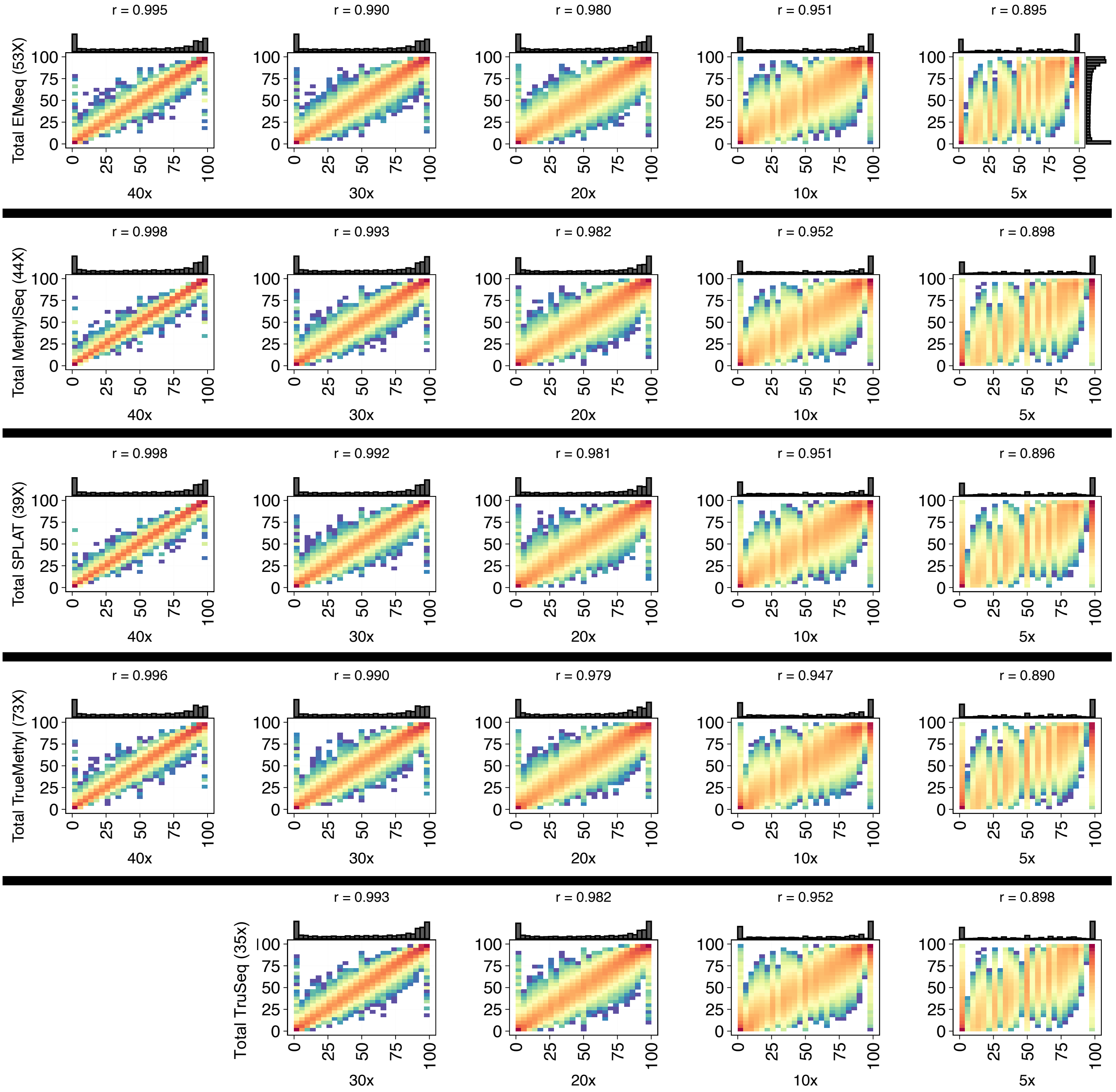

Figure S9: Pearson correlations of methylation percentage estimation within each assay, comparing the total data (y- axes) against their respective downsampled schema (x-axes), for combined replicates of HG002 libraries. Pearson values are shown above each comparison, as well as marginal histograms showing methylation percentage distributions. For TruSeq, the total data returned a mean coverage of 35X, meaning that a comparison to 40X downsampling was not possible.

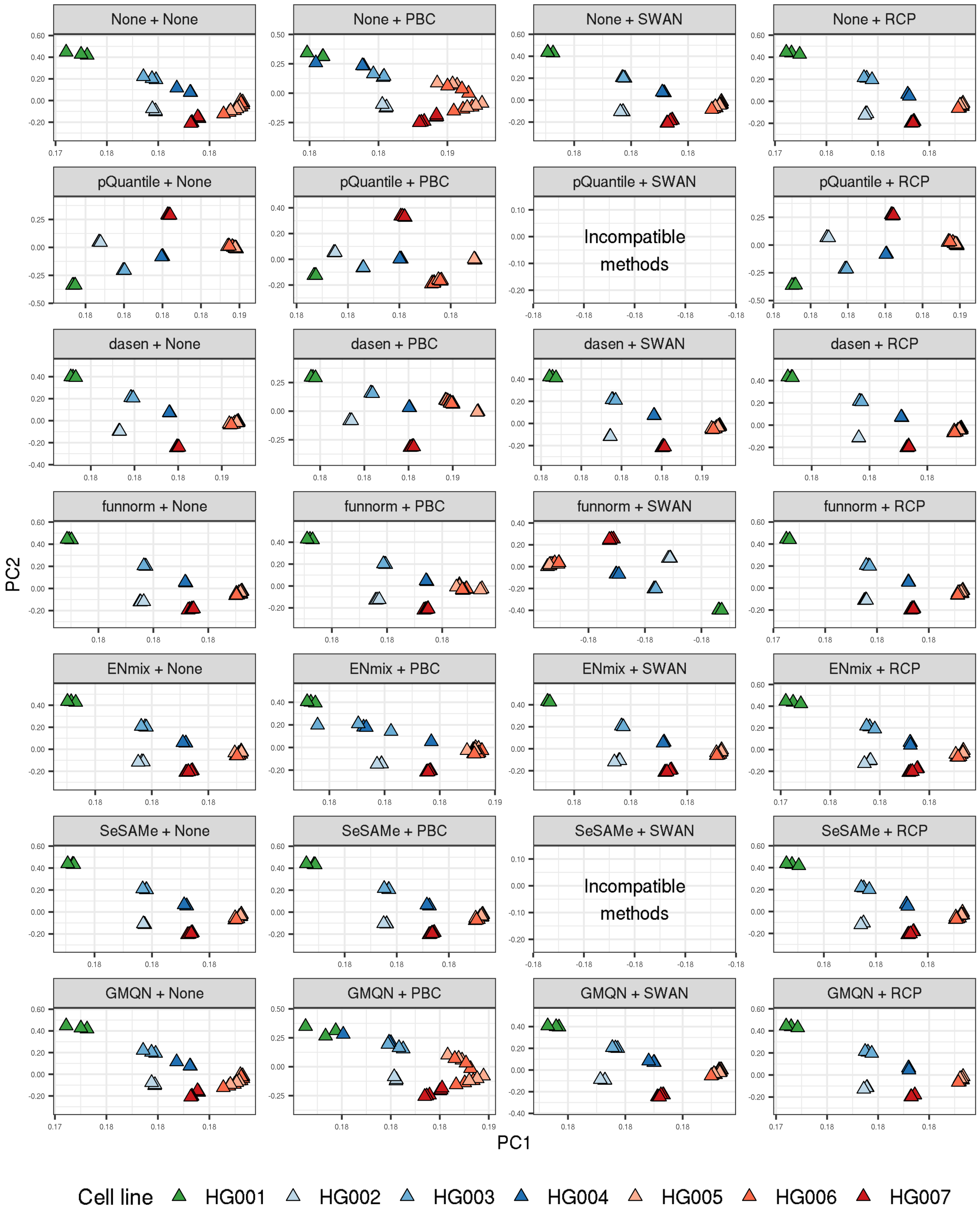

Figure S10: First two principal components (PCs) calculated from 678,597 CpG sites with complete information in all normalized microarray datasets, by normalization pipeline.

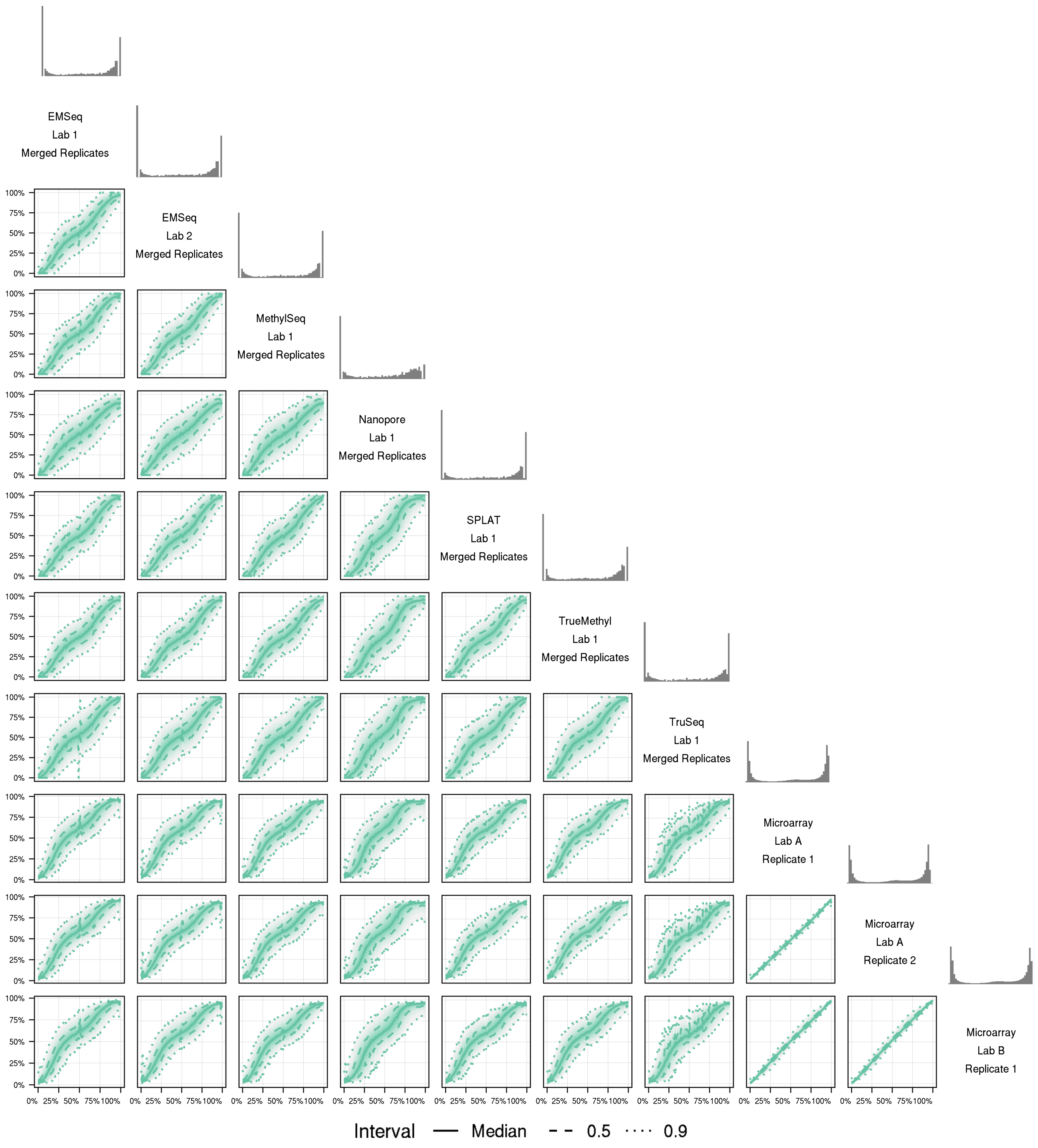

Figure S11: Distribution of beta values across HG002 samples at 841,833 CpG sites with complete information in all assays. Beta values for the assay on the x axis were binned (binwidth=0.01) to calculate beta value deciles for the assay on the y axis, indicated by the color transparency. 90% of the y-axis values fall between the outermost dotted lines for each bin along the x-axis. Marginal histograms for each assay are shown above the assay label.

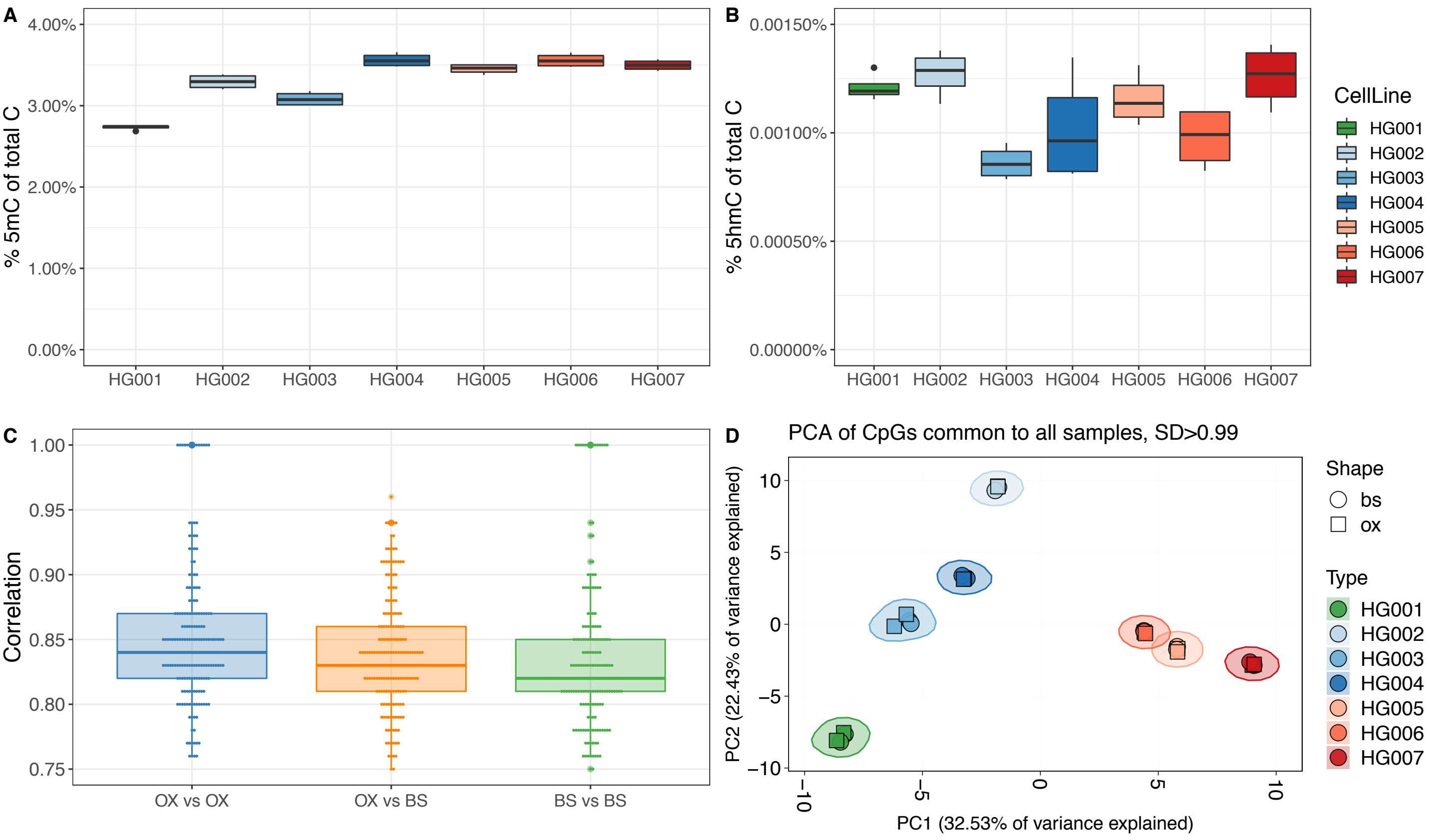

Figure S12: Capture of 5mC and 5hmC from TrueMethyl replicates, including bisulfite-only (bs) and oxidative bisulfite (ox). (a) Percent of inferred 5mC among all cytosines in the genome. (b) Percent of inferred 5hmC among all cytosines in the genome. (c) Pearson correlation of replicates across genomes between oxidative and bisulfite replicates. (d) Unsupervised clustering of samples, including OX and BS samples.

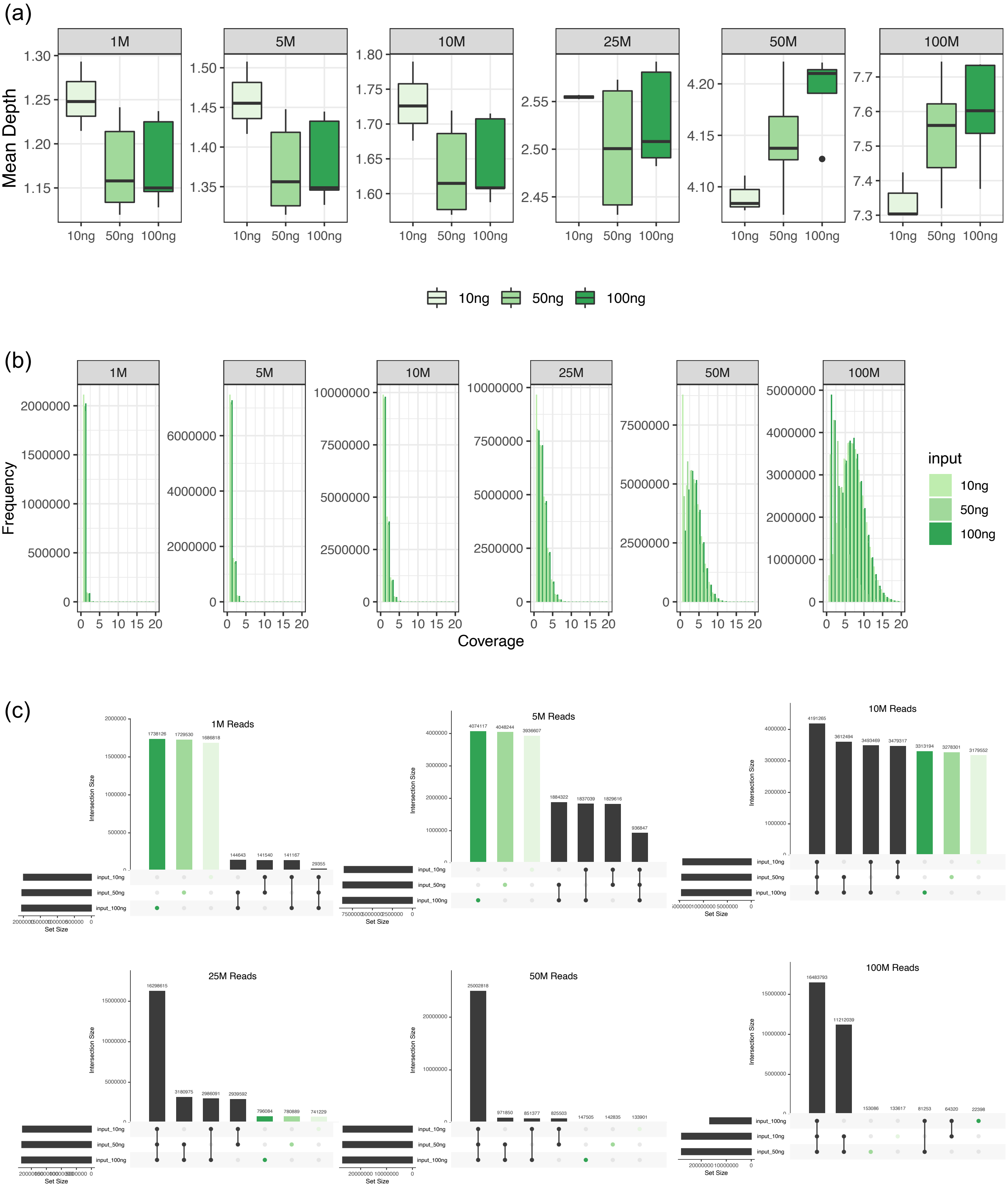

Figure S13: EM-Seq read titration experiment. Replicates generated using 10ng, 50ng, and 100ng of input DNA for HG005, HG006, and HG007 were randomly downsampled to 1M, 5M, 10M, 25M, 50M, and 100M paired end 150bp input reads. (a) Distribution of mean depth of CpGs covered for each input amount. (b) Read coverage distributions per input type per downsampled read amount. (c) UpSet plots showing the intersections of CpGs shared by each downsampling scheme, as well as uniquely covered CpGs.

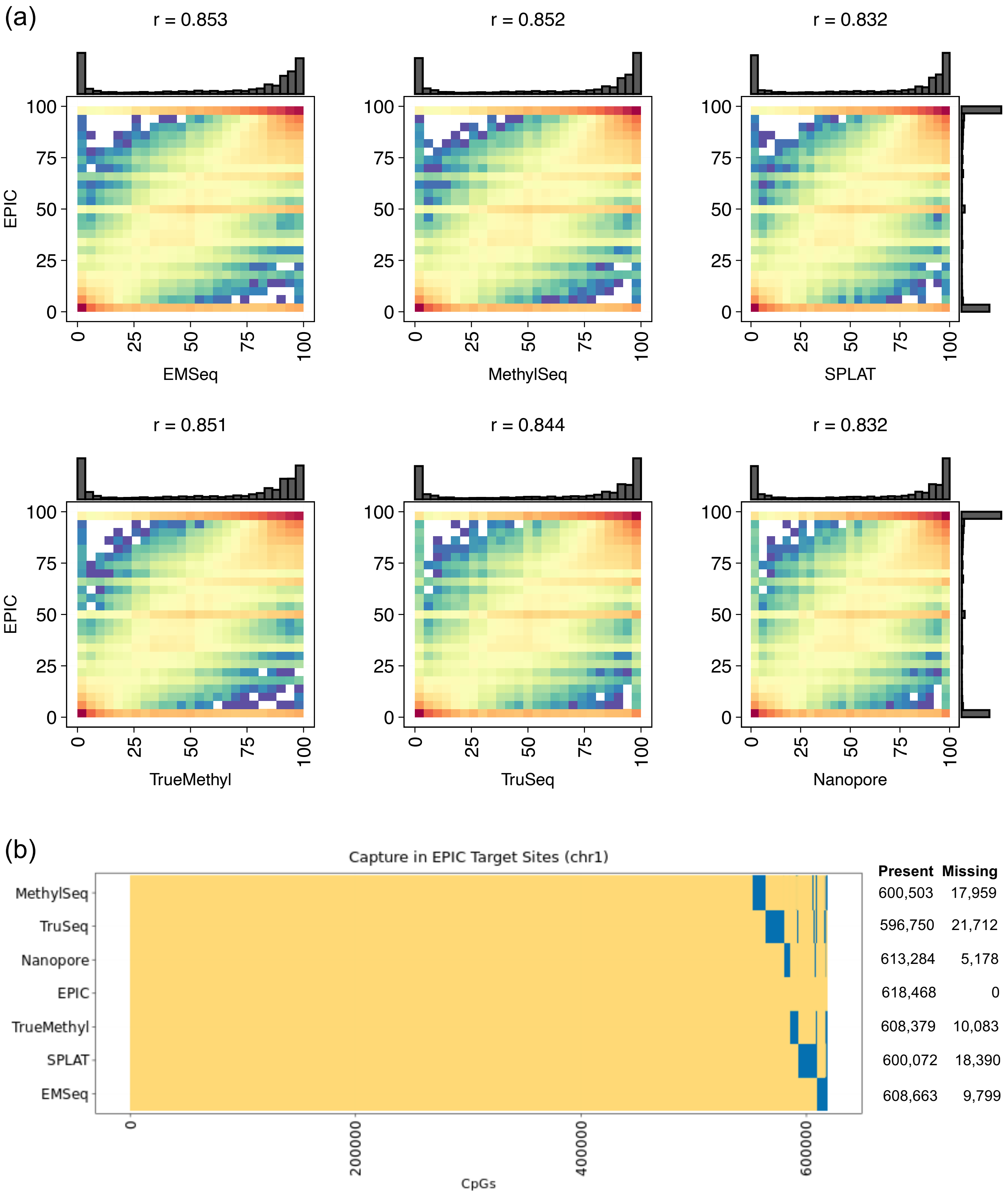

Figure S14: (a) Pearson correlation of percent methylation estimates of Methyl Seq EPIC Capture versus each whole methylome library. All values are shown for Chromosome 1 of HG002 replicates. (b) Distribution of CpGs covered (in yellow) or missed (in blue) by each assay on Chromosome 1. Total values are shown per assay in the table on the right.
