# Supplementary material for: The SEQC2 epigenomics quality control (EpiQC) study
Source: Genome Biol. 2021 Dec 6;22:332. doi: 10.1186/s13059-021-02529-2 (PMC8650396; doi:10.1186/s13059-021-02529-2)
Supplement: Supplementary file 2 — Additional file 2. contains the supplementary tables (Supplementary Table 1–6). [file 13059_2021_2529_MOESM2_ESM.pdf]

Supplementary Tables

Step	Software	No. Reads	Mean Time (min)	Standard Dev (min)
Alignment	Bismark	1M	19.33	1.74
	BitmapperBS	1M	11.98	3.76
	BSseeker2	1M	65.76	3.5
	bwa-meth	1M	29.92	6.17
Meth Calling	Bismark	1M	2.86	0.9
	BitmapperBS	1M	1.97	0.17
	BSseeker2	1M	39.87	17.4
	bwa-meth	1M	0.24	0.07

Supplementary Table 1. Timing comparison of each alignment and methylation estimation software. Ten permutations of each software were run, and the mean times are reported, as well as the standard deviation per software.

Insert Size	HG001	HG002	HG003	HG004	HG005	HG006	HG007	mean	stDev
EMSeqLAB01	297.1	299.6	349.75	288.95	300.53	298.23	330.3	309.21	22.11
EMSeqLAB02	325.8	327.95	326.35	328.15	320.2	325.47	358.57	330.35	12.72
MethylSeq	249.77	250.93	250.88	247.13	249.67	256.45	247.78	250.37	3.04
SPLAT	214.83	217.81	220.71	223.74	220.34	223.85	224.18	220.78	3.52
TruSeq	218.58	217.68	219.88	216.77	214.67	217.58	215.78	217.28	1.74
TrueMethylOX	207.7	203.83	209.95	205.93	211.8	211.5	204.4	207.87	3.3
TrueMethylBS	228.6	220.67	226.5	224.2	224.73	227.4	219	224.44	3.52
EPIC	225.7	227.45	225.65	227	229.65	229.7	227.7	227.55	1.65
**Primary Mapping %**	**HG001**	**HG002**	**HG003**	**HG004**	**HG005**	**HG006**	**HG007**	**mean**	**stDev**
EMSeqLAB01	97.48	97.63	97.37	97.87	97.14	97.36	97.32	97.45	0.24
EMSeqLAB02	94.89	93.27	94.09	94.46	91.97	94.27	93.33	93.75	0.98
MethylSeq	98.4	98.36	98.32	98.41	98.16	98.36	98.39	98.34	0.09
SPLAT	96.44	97.18	96.92	97.21	97.07	97.48	97.24	97.08	0.33
TruSeq	95.33	95.45	95.23	95.58	95.51	95.55	95.37	95.43	0.13
TrueMethylOX	85.95	84.53	87.43	87.01	85.64	87.15	85.11	86.12	1.11
TrueMethylBS	85.17	83.5	86.66	86.06	84.73	86.4	84.2	85.25	1.18
EPIC	98.27	98.31	98.37	98.12	98.28	98.27	98.23	98.26	0.08
**Duplicate %**	**HG001**	**HG002**	**HG003**	**HG004**	**HG005**	**HG006**	**HG007**	**mean**	**stDev**
EMSeqLAB01	9.28	9.13	9.15	8.65	11.28	12.1	10.6	10.03	1.3
EMSeqLAB02	23.61	25.03	23.97	23.37	27.08	23.68	25.11	24.55	1.31
MethylSeq	13.75	13.77	13.84	14.42	13.56	14.44	13.32	13.87	0.42
SPLAT	12.37	12.02	11.63	10.9	11.88	10.86	13.28	11.85	0.84
TruSeq	21.73	22.53	27.26	24.35	25.86	31.57	25.77	25.58	3.28
TrueMethylOX	21.24	21.66	18.19	19.48	20.86	19.56	21.21	20.31	1.26
TrueMethylBS	21.29	21.95	17.89	18.72	20.15	18.66	20.22	19.84	1.48
EPIC	66.42	67.43	62.9	63.92	69.21	69.5	68.24	66.8	2.56
**Dinucleotide Bias**	**HG001**	**HG002**	**HG003**	**HG004**	**HG005**	**HG006**	**HG007**	**mean**	**stDev**
EMSeqLAB01	3.77	2.97	3.36	2.98	2.7	2.69	2.99	3.07	0.38
EMSeqLAB02	1.12	0.84	1.16	0.98	1.14	1.11	1.1	1.06	0.11
EPIC	26.58	26.74	26.59	26.69	26.43	26.36	26.93	26.62	0.19
MethylSeq	3.4	3.71	3.43	3.41	3.47	3.53	3.52	3.5	0.11
SPLAT	6.95	7.58	5.55	6.36	6.66	6.24	5.8	6.45	0.69
TrueMethylBS	3.43	3.44	3.8	3.3	3.73	3.61	3.99	3.61	0.24
TrueMethylOX	3.3	3.71	3.64	3.71	3.72	3.72	3.71	3.64	0.16
TruSeq	24.66	23.09	24.1	23.69	23.13	23.36	23.83	23.69	0.56
**Useable Bases %**	**HG001**	**HG002**	**HG003**	**HG004**	**HG005**	**HG006**	**HG007**	**mean**	**stDev**
EMSeqLAB01	90.21	90.35	90.26	90.71	87.88	87.61	88.89	89.42	1.27
EMSeqLAB02	76.01	74.6	75.51	76.16	72.62	76.01	51.56	71.78	9
EPIC	33.2	32.21	36.69	35.67	30.46	30.17	31.4	32.83	2.52
MethylSeq	74.46	74.5	74.55	73.8	74.77	74.01	74.9	74.43	0.39
SPLAT	78.9	80.62	81.19	83	81.43	82.79	81.4	81.33	1.38
TrueMethylBS	70.21	66.6	71.08	70.13	70.03	71.69	71.97	70.24	1.78
TrueMethylOX	67.53	66.49	68.45	68.5	68.07	68.66	66.78	67.78	0.87
TruSeq	62.68	62.98	60.23	61.19	60.29	62.58	51.03	60.14	4.17

Supplementary Table 2 Read and mapping statistics for all cell lines. stDev = standard deviation. Values shown are averages across replicates for each library. Usable bases are calculated as the total mapped bases as a percentage of the total number of bases sequenced.

Number of Common Sites for all Assays	2298846	Assay					
Common Sites with 5X for all Assays (C5X Sites)	1928536						
DM Sites in 3 or more platforms on C5X sites (DM4+)	29802	EM-Seq	Methyl-Seq	Nanopore	SPLAT	TrueMethyl	TruSeq
Percentage of all common sites with 5X Coverage		97%	96%	100%	96%	97%	86%
Number of DM Sites for this assay on C5X Sites (DMA)		74054	67621	26868	76591	59516	87170
Percentage DMA unique to this platform		26%	26%	17%	29%	22%	42%
Percentage of DMA sites in DM4+		36%	38%	56%	35%	42%	27%
Percentage of DM4+ in DMA sites		90%	86%	51%	89%	85%	79%

Supplementary Table 3 Statistics for differentially methylated sites across assays.

	EMSeq	MethylSeq	Nanopore	SPLAT	TrueMethyl	TruSeq
Number of DMAs mapped to array	3296	2964	1027	3228	2750	4267
Number DMAs with |PMD| > .2	3279	2942	1026	3092	2743	3404
% DMAs with |PMD| >.2 and array |PMD| > .2	55.5%	58.8%	67.0%	57.3%	60.0%	49.6%
Number Hypermethylated in HG005-HG007	2505	2358	721	2368	2092	2432
% Hymermethylated DMAs with array PMD > .2	57.0%	60.3%	69.1%	58.6%	62.0%	52.4%
Number Hypomethylated in HG005-HG007	774	584	305	724	651	972
% Hypomethylated DMAs with array PMD < -.2	50.4%	53.1%	62.0%	53.0%	53.8%	42.7%
% of sites with array |PMD|>.2 identified as DMAs	44.0%	41.9%	16.6%	43.5%	39.8%	42.7%

Supplementary Table 4 Concordance between assays of differentially methylated sites per assay (DMAs) with respect to microarray sites. PMD = Percent Methylation Difference, calculated as an absolute value.

TargetID	African American	Caucasian American	Asian American	Asian-Caucacian	FDR	Chr.	Position (HG19)	Position (HG38)	Gene	Feature	Variance	meQTL	EMSeq	MethylSeq	Nanopore	SPLAT	TrueMethyl	TruSeq	Illumina
cg16590012	0.84	0.85	0.62	-0.23	2.73E-29	1	1265354	1329974	TAS1R3	Promoter	AS	yes	-0.7629	-0.6364	-0.3709	-0.6693	-0.6275	-0.399	-0.2610
cg23611477	0.89	0.81	0.75	-0.06	4.11E-11	1	1644835	1713396	CDK11A;CDK11B	Body;Promoter	AF	yes	NA	0.4202	0.0614	NA	NA	NA	0.0538
cg00669623	0.28	0.15	0.14	-0.01	2.76E-17	1	1655867	1724428	CDK11B;CDK11A	Promoter	AF	no	-0.0133	0.0033	0.013	0.0111	0.0147	-0.018	0.0057
cg03396347	0.73	0.60	0.61	0.01	1.08E-05	1	1875803	1944364			AF	yes	-0.0497	-0.1173	0.0269	-0.239	0.0752	-0.04	-0.0173
cg00095688	0.62	0.66	0.52	-0.14	1.24E-05	1	2003864	2072425	PRKCZ	Promoter	AS	no	-0.0687	-0.0143	-0.0531	-0.2671	-0.1292	-0.0435	-0.0499
cg10761639	0.74	0.84	0.69	-0.15	1.09E-17	1	2023794	2092355	PRKCZ	Promoter	CA	yes	0.0025	0.0335	-0.1343	-0.0615	-0.0867	-0.0412	-0.0425
cg24499605	0.45	0.32	0.43	0.11	1.76E-14	1	3142925	3226361	PRDM16	Body	CA	no	0.061	0.0478	0.1165	0.0104	-0.0335	0.129	0.0281
cg14654471	0.91	0.89	0.75	-0.14	3.57E-14	1	5937169	5877109	NPHP4	Body	AS	yes	-0.0249	0.0024	-0.0453	0.0282	-0.0055	0.0189	-0.1263
cg13549940	0.64	0.81	0.81	0.00	2.30E-12	1	6390053	6329993	ACOT7	Body	AF	yes	-0.2485	-0.1441	-0.214	-0.1369	-0.0986	-0.1721	-0.1473
cg23914842	0.32	0.39	0.50	0.11	1.21E-07	1	9327170	9267111	H6PD	3'UTR	AS	yes	-0.0111	-0.1046	-0.0248	-0.2128	-0.1016	-0.0768	0.0269
cg01017257	0.57	0.48	0.61	0.13	3.62E-05	1	15059738	14733242	KIAA1026/KAZN	Body;Body	CA	yes	0.7907	0.649	0.6319	0.6125	0.5573	0.617	0.6381
cg04850659	0.31	0.26	0.40	0.14	1.05E-08	1	17019133	16692638	ESPNP	Body	AS	no	-0.1329	NA	-0.0215	-0.0769	NA	-0.0618	-0.0007
cg16558994	0.30	0.21	0.36	0.15	2.37E-05	1	21023132	20696639	KIF17	Body	CA	yes	0	0.0968	0	NA	-0.0179	0.0052	-0.0176
cg18150584	0.57	0.50	0.64	0.14	6.28E-04	1	23887816	23561326	ID3	Promoter	CA	no	0.083	0.0777	0.025	0.2504	0.0816	0.2045	0.1313
cg19276111	0.43	0.55	0.49	-0.06	2.33E-03	1	24229232	23902742	CNR2	Promoter	AF	no	-0.0678	0.0343	-0.14	-0.0744	-0.1275	-0.0814	-0.2102
cg20415053	0.54	0.62	0.74	0.12	1.60E-05	1	26527928	26201437	CATSPER4	Body	AS	yes	0.0882	0.0313	0.1282	0.2414	0.0432	0.1908	0.1243
cg02251754	0.50	0.29	0.18	-0.11	3.50E-20	1	28572299	28245788			AF/AS	yes	-0.3134	-0.3603	-0.4901	-0.4694	-0.4127	-0.4026	-0.4259
cg14781242	0.66	0.81	0.84	0.03	9.37E-14	1	32738251	32272650	LCK	Promoter	AF	yes	0.0052	-0.1846	0.0507	0.0287	-0.1409	-0.0417	-0.0160
cg06917450	0.29	0.27	0.54	0.27	2.31E-16	1	38156652	37690980	C1orf109	Promoter	AS	yes	0.5083	0.5241	0.4751	0.439	0.4331	0.4884	0.5093
cg26038582	0.69	0.57	0.64	0.07	1.73E-02	1	42384390	41918719	HIVEP3	Promoter	CA	no	-0.2774	-0.185	-0.0661	0.021	-0.234	0.131	-0.2571
cg02927682	0.37	0.40	0.49	0.09	1.75E-03	1	54844424	54378751	SSBP3	Body	AS	yes	0.2256	0.1371	0.105	0.1135	0.138	0.2669	0.2199
cg10760651	0.48	0.37	0.50	0.13	1.06E-04	1	86968184	86502501			CA	yes	0.2962	0.3508	0.346	0.323	0.2832	0.2422	0.1705
cg10631373	0.41	0.29	0.36	0.07	2.25E-04	1	89457642	88991959	RBMXL1;CCBL2	Promoter;Promoter	CA	yes	0.1154	0.0038	0.0936	0.1048	0.1652	-0.0228	0.1258
cg09408571	0.59	0.66	0.75	0.09	5.84E-07	1	101003634	100538078	GPR88	Promoter	AF	yes	0.1117	0.0329	-0.0697	-0.0308	0.2187	0.0961	0.0979
cg06223162	0.30	0.38	0.53	0.15	5.65E-08	1	101003688	100538132	GPR88	Promoter	AS	yes	0.1265	0.1352	0.1149	0.132	0.0938	0.1667	0.1493
cg25210835	0.25	0.28	0.46	0.18	2.81E-09	1	110254828	109712206	GSTM5	Promoter	AS	yes	-0.4127	-0.1016	-0.208	0.0643	-0.0629	-0.2639	-0.1339
cg02193146	0.64	0.79	0.76	-0.03	6.37E-06	1	110752257	110209635		ncRNA promoter	AF	no	0.1198	0.0101	0.0234	0.0045	-0.075	-0.0532	-0.0285
cg24853868	0.51	0.49	0.66	0.17	2.26E-05	1	146555624	147084075			AS	yes	0.3135	0.5334	0.1769	0.2033	0.1937	0.2011	0.1400
cg13502125	0.66	0.63	0.77	0.14	8.15E-05	1	147826191	148354063			AS	yes	-0.0759	-0.1163	-0.0008	0.0058	-0.0483	0.1103	-0.0616
cg09359103	0.45	0.41	0.22	-0.19	7.67E-15	1	154839909	154867433	KCNN3	Body	AS	yes	-0.5463	-0.5508	-0.4614	-0.541	-0.4958	-0.5729	-0.5778
cg23915527	0.50	0.36	0.39	0.03	2.45E-05	1	161368787	161398997			AF	yes	0.2098	0.2852	0.1048	0.1548	0.0742	0.2871	0.2215
cg12092579	0.38	0.23	0.29	0.06	2.07E-06	1	178380975	178411840	RASAL2	Body	AF	no	-0.2273	-0.3367	-0.0571	-0.3965	-0.4271	-0.3319	-0.3504
cg21868798	0.36	0.30	0.24	-0.06	3.09E-05	1	199481399	199512271			AF	yes	0.2709	0.1163	-0.0715	0.1599	0.1324	0.1697	0.1315
cg18222590	0.41	0.35	0.48	0.13	1.21E-10	1	204290972	204321844	PLEKHA6	Promoter	CA	yes	0.1774	0.1619	0.0542	0.1112	0.1024	0.1	0.1857
cg20240347	0.46	0.31	0.35	0.04	1.72E-04	1	204465584	204496456			AF	yes	0.0271	-0.0594	-0.1258	0.029	-0.0769	0.1378	0.0815
cg17178900	0.28	0.50	0.24	-0.26	2.76E-10	1	205818956	205849828	PM20D1	Body	CA	yes	-0.4676	-0.555	-0.4076	-0.4483	-0.5194	-0.6068	-0.5164
cg26354017	0.31	0.50	0.28	-0.22	1.98E-08	1	205819088	205849960	PM20D1	Promoter	CA	yes	-0.5308	-0.4969	-0.4403	-0.6205	-0.5653	-0.6419	-0.4909
cg14159672	0.30	0.48	0.26	-0.22	5.54E-11	1	205819179	205850051	PM20D1	Promoter	CA	yes	-0.6875	-0.6542	-0.643	-0.5692	-0.6211	-0.6649	-0.6134
cg14893161	0.26	0.38	0.22	-0.16	2.00E-11	1	205819251	205850123	PM20D1	Promoter	CA	yes	-0.5273	-0.4989	-0.5044	-0.5009	-0.3619	-0.4667	-0.4443
cg11965913	0.15	0.30	0.11	-0.19	9.61E-14	1	205819406	205850278	PM20D1	Promoter	CA	yes	-0.1019	-0.2599	-0.1607	-0.1985	-0.2434	-0.2879	-0.2005
cg24503407	0.25	0.43	0.21	-0.22	1.11E-13	1	205819492	205850364	PM20D1	Promoter	CA	yes	-0.6528	-0.5719	-0.5636	-0.5621	-0.6911	NA	-0.5648
cg07157834	0.33	0.46	0.28	-0.18	2.78E-09	1	205819609	205850481	PM20D1	Promoter	CA	yes	-0.5907	-0.6364	-0.4552	-0.5556	-0.6012	-0.3885	-0.5779
cg06935979	0.62	0.48	0.46	-0.02	1.01E-06	1	232941706	232805960	KIAA1383/MAP10	Promoter	AF	yes	0.1941	0.1563	0.2429	0.1696	0.3363	0.2297	0.1942
cg00951395	0.49	0.32	0.33	0.01	7.12E-09	1	232941775	232806029	KIAA1383/MAP10	Promoter	AF	yes	0.0731	-0.0597	-0.1212	0.071	-0.0593	0.0451	0.0826
cg02889973	0.46	0.39	0.51	0.12	3.25E-04	1	234977572	234841825			CA	no	0.2911	0.332	0.1873	0.3361	0.3551	0.1044	0.3384
cg09033006	0.40	0.46	0.22	-0.24	5.74E-19	1	244517177	244353875	C1orf100	Promoter	AS	yes	-0.1061	-0.1756	-0.1901	-0.206	-0.2146	-0.2098	-0.2268
cg19368911	0.61	0.70	0.75	0.05	1.01E-07	1	245541456	245378154	KIF26B	Body	AF	no	0.2452	0.0935	0.2145	0.2595	0.1635	0.0662	0.1769
cg04134399	0.28	0.15	0.28	0.13	9.18E-09	1	246231142	246067840	SMYD3	Body	CA	no	-0.082	-0.0981	0.0651	-0.066	-0.0339	-0.14	-0.0326
cg04798314	0.51	0.66	0.84	0.18	2.26E-13	1	246668601	246505299	SMYD3	Body	AS	yes	-0.0724	0.1592	-0.0085	-0.0506	-0.0733	0.0421	-0.0321
cg09226051	0.42	0.40	0.30	-0.10	4.03E-03	1	247611502	247448200	NLRP3	Body	AS	yes	-0.0714	-0.154	-0.017	-0.1633	0.034	-0.0239	-0.2073
cg15829088	0.33	0.37	0.45	0.08	3.36E-04	1	247802935	247639633		ncRNA promoter	AS	yes	0.0851	-0.0693	0.0111	-0.2654	0	-0.0899	-0.0053

Supplementary Table 5 Population Variance agreement. A total of 52 CpGs on chromosome 1 that had been identified as differentially methylated between ethnic populations were annotated and compared for concordance of differential signal between microarray and sequencing data.

fmoles of different forms identified

		Name	dC	dmC	dmC	dhmC	dmC/dC	dhmC/dmC	dhmC/dC
Biological Replicate 1	HG001	CP_01	5680.727	156.3333	2143.964	0.922163	2.75%	0.04%	0.0012%
		CP_01	5721.555	157.2296	2212.077	0.966647	2.75%	0.04%	0.0012%
	HG002	CP_02	6134.437	206.1124	2915.523	1.078334	3.36%	0.04%	0.0012%
		CP_02	6097.877	206.3662	2893.051	0.969016	3.38%	0.03%	0.0011%
	HG003	CP_03	6676.031	212.3023	2979.854	0.756934	3.18%	0.03%	0.0008%
		CP_03	6742.651	211.5162	2948.914	0.739288	3.14%	0.03%	0.0008%
	HG004	CP_04	5223.132	188.2691	2588.429	0.592667	3.60%	0.02%	0.0008%
		CP_04	5224.774	191.126	2560.265	0.56839	3.66%	0.02%	0.0008%
	HG005	CP_05	5487.878	192.3814	2680.448	0.828852	3.51%	0.03%	0.0011%
		CP_05	5523.128	193.3392	2646.98	0.784192	3.50%	0.03%	0.0010%
	HG006	CP_06	5962.204	217.7679	2979.255	0.724408	3.65%	0.02%	0.0009%
		CP_06	6041.553	217.7672	3002.681	0.686946	3.60%	0.02%	0.0008%
	HG007	CP_07	5819.142	205.9319	2860.277	0.884028	3.54%	0.03%	0.0011%
		CP_07	5733.883	204.7114	2814.214	0.937631	3.57%	0.03%	0.0012%
Biological Replicate 2	HG001	CP_08	3620.176	99.23043	1362.303	0.646334	2.74%	0.05%	0.0013%
		CP_08	3674.493	98.73453	1356.403	0.582917	2.69%	0.04%	0.0012%
	HG002	CP_09	2835.62	91.63515	1259.922	0.537728	3.23%	0.04%	0.0014%
		CP_09	2872.229	92.00553	1250.31	0.520239	3.20%	0.04%	0.0013%
	HG003	CP_10	2832.307	85.18032	1167.688	0.370097	3.01%	0.03%	0.0010%
		CP_10	2864.241	86.2764	1175.466	0.351737	3.01%	0.03%	0.0009%
	HG004	CP_11	2987.18	104.5185	1423.736	0.548236	3.50%	0.04%	0.0013%
		CP_11	2989.671	104.0718	1431.635	0.452427	3.48%	0.03%	0.0011%
	HG005	CP_12	2999.949	102.7485	1410.854	0.489238	3.43%	0.03%	0.0012%
		CP_12	3048.796	103.0123	1399.723	0.54343	3.38%	0.04%	0.0013%
	HG006	CP_13	3258.307	113.3027	1535.199	0.48512	3.48%	0.03%	0.0011%
		CP_13	3199.5	111.8209	1519.138	0.476289	3.49%	0.03%	0.0011%
	HG007	CP_14	3324.166	113.9253	1545.66	0.634276	3.43%	0.04%	0.0014%
		CP_14	3266.074	112.9173	1530.64	0.60005	3.46%	0.04%	0.0014%
Blank		CP_15	0		0.681001				
		CP_15	0		0.689081				

Supplementary Table 6 LC-MS/MS quantification of dC, dmC and dhmC in fmoles from digested genomic DNA (HG001-HG007) samples. For the detection of 5hmC a second higher volume injection was performed. The two dmC quantification values correspond to the two injections. Percentage of 5hmC in these samples is very low and below the limit of detection of the method.
