# Supplementary material for: Author Correction: The SEQC2 epigenomics quality control (EpiQC) study
Source: Genome Biol. 2021 Dec 23;22:350. doi: 10.1186/s13059-021-02573-y (PMC8705091; doi:10.1186/s13059-021-02573-y)
Supplement: Supplementary file 1 — Additional file 1. Contains the supplementary figures (Supplementary Figure 1–14). [file 13059_2021_2573_MOESM1_ESM.pdf]

Supplementary Methods

Whole Methylome Sequencing Across Centers

Short-read sequencing details: The short-read sequencing libraries were collected from participating laboratories and sequenced centrally on NovaSeq 6000 systems at one or two sequencing centers. Libraries were pooled by library type in high concentration equimolar stock pools (4 nM). After pooling, bead-based clean-up was performed to remove peaks <200 bp. Briefly, 0.7 X volume of NEBNext Sample Purification beads was added to the pools and incubated for 10 mins at room temperature. The beads were clarified by placing on a magnet and washed twice with freshly prepared 80% ethanol. Beads were allowed to dry for 2 mins and resuspended in 0.1 X TE. The cleaned stock pools were quantified on an Agilent Bioanalyzer using High sensitivity DNA chip.

Sequencing Center 1: Pooled libraries were diluted to 1.5 nM. were loaded on a NovaSeq S4 flowcell with a final loading concentration of 250 pM for all libraries with the exception of EM-Seq, which was loaded at 300 pM. Unrelated standard libraries were added at 5% instead of PhiX to balance the base composition during sequencing. All libraries were sequenced PE150 according to the manufacturer's instructions (Illumina) with targeted per replicate CG coverage of 20x.

Base calling was performed using RTA v3.4.4 In cases where libraries were not prepared with dual-unique indices, they were demultiplexed using the expected index 2 sequence derived from the universal adapter. Demultiplexing and fastq generation was performed using Picard 2.20.6 using default settings except as listed below:

```
picard ExtractIlluminaBarcodes MAX_NO_CALLS=0 MIN_MISMATCH_DELTA=2
MAX_MISMATCHES=2 picard IlluminaBasecallsToFastq \
    read_structure=100T8B8B100T RUN_BARCODE=A00336 \
    LANE=<lane> FIRST_TILE=<tile> TILE_LIMIT=1 \
    MACHINE_NAME=<instrument> FLOWCELL_BARCODE=<flowcell>
```

Sequencing Center 2: The high concentration equimolar stock library pools were sent to Illumina in order to ameliorate depth of sequencing for the WGBS libraries. Libraries pools were diluted to 1.5 nM and a final loading concentration of 300 pM was loaded on the flow cell with 5% PhiX. The libraries were sequenced on an Illumina NovaSeq 6000 S4 flowcell with direct flow cell loading (XP workflow) according to manu facturer's instructions. MethylSeq, SPLAT and TruSeq pools were multiplexed on two lanes; SPLAT libraries on their own in the third lane; and TrueMethyl libraries on their own in the fourth lane. Base calling was performed using RTA v3.4.4. Run data were uploaded to BaseSpace and fastq files were generated using default parameters.

Supplementary Results

Alignment and Methylation Caller Comparisons

The first step after data QC was to map reads to a reference genome and estimate levels of methylation per CpG. We evaluated the performance of commonly used alignment/methylation calling packages, including Bismark [1], BitMapperBS [2], BSseker2 [3], bwa-meth [4], and gemBS [5]. For each software, we aligned reads to the GRCh38 human reference genome, with a set of bisulfite controls appended as additional contigs (see methods and **Additional file 1: Figure S2**). We focused our analysis on Ashkenazi Son (HG002) data for these comparisons, using all replicates from each of the five short read epigenetic library types.

Although we successfully ran gemBS, its outputs were removed from further comparison for two reasons: (1) the maximum likelihood-based modeling of methylation percentages did not allow for merging of values across replicates, and (2) an unusually low percentage of CpGs were detected compared to all other platforms, prohibiting genome-wide comparison.

The mapping of reads showed aligner-specific distributions (**Additional file 1: Figure S3a**). bwa-meth was able to map the highest percentage of reads to the reference genome, followed by bitmapperBS, BSSeeker2, and then Bismark. bwa meth and Bismark tend to allow reads to align to multiple locations in the genome (marking these reads as secondary or supplementary alignments and ignoring them for methylation calling). BitMapperBS and BSseeker2 more commonly kept reads unmapped rather than align them ambiguously, although Bismark had the highest rate of unmapped reads. All four softwares had similar rates of duplicate read marking, except for BSseeker2 which tended to mark fewer reads as duplicates. It should be noted that an external program, Picard MarkDuplicates was used for deduplication in bwa-meth, BitMapperBS, and BSseeker2. Despite this, BSseeker2 samples still had fewer duplicate reads than other library types.

We then calculated the mapping efficiency, defined as the percentage of bases aligned and retained for methylation calling (see below for the effects of read filtration) divided by the total bases per replicate (**Additional file 1: Figure S3b**), as well as the mean coverage achieved per CpG dinucleotide (**Additional file 1: Figure S3c**). bwa-meth returned both the most efficient mapping rate, as well as the highest mean coverage per CpG within every dataset except for TruSeq, where outputs from each software matched very closely. Generally, BitMapperBS scored second in efficiency and depth of coverage, followed by Bismark, then BSseeker2.

The running time of each aligner was tested using one million random paired-end reads from each replicate and run ten times, summarized in Supplementary Table 1. BitMapperBS was the fastest aligner, with an average of 11.98 minutes required to align 1M paired end reads. This was followed by Bismark, then bwa-meth, then BSseeker2 requiring significantly more time than the other three. For methylation calling, bwa-meth (leveraging MethylDackel) was by far the fastest, requiring 0.24 minutes on average.

We then tested the distribution of CpGs called by each software (**Additional file 1: Figure S3d**) to look for

any aligner-specific biases. All four programs returned a nearly identical distribution of CpGs called throughout the genome. The highest genomic enrichment was detected at 5'UTRs, promoter regions, and exonic regions by all programs. Therefore, even though mapping efficiency and CpG depth was influenced by software, the genomic distribution of CpGs was reliably called by all softwares examined. As a result of these comparisons, outputs from bwa-meth were used for all downstream analyses.

5-hydroxymethylcytosine Detection

Total 5-methylcytosine (5mC) and 5-hydroxymethylcytosine (5hmC) levels within each cell line examined in this study were measured by LC-MS/MS (Supplementary Table 6). The estimated percentage of 5hmC levels across all seven cell lines were below the limit of detection for this method.

In order to validate these results at base-level resolution, we used the NuGEN TrueMethyl oxBS-Seq library preparation kit (aka TrueMethyl), which allows investigators to measure 5mC and 5hmC in an indirect manner on the sequence level. For completeness, each cell line replicate was processed using both bisulfite only (BS = 5mC + 5hmC) and an oxidative reaction prior to sodium bisulfite treatment (OX = 5mC only).

Additional file 1: Figure S12 shows that all cell lines have a higher level of 5mC compared to 5hmC (**Additional file 1: Figure S12a,b**). The low 5hmC levels were also observed at the single-nucleotide resolution level, with similar correlations between the two library preparations across all cell lines (**Additional file 1: Figure S12c**), and also within each cell lines (**Additional file 1: Figure S12d**), where the PCA plot shows little to no separation between libraries prepared using BS or OX protocols.

As stated above, preparation of BS and OX libraries in parallel allows the determination of 5mC, 5hmC and C. We used the MLML2R package to estimate the level of each cytosine state, for each CpG sequenced, using HG002 as example (**Additional file 1: Figure S12e**). The top panel shows that some CpG sites not only show 100% of a specific cytosine mark (C = 100% unmethylated CpG, mC = 100% methylated CpG), but also a mixture of two (mC_C = methylated or unmethylated C; hmC_C = hydroxymethylated or unmethylated C; mC_hmC = methylated or hydroxymethylated C) or of all cytosine mark (mC_hmC_C). Consistent with the LC-MS/MS quantitation, hmC marks were found in low proportions at some CpG sites. The results observed for HG002 were representative of all the 7 cell lines.

Biological Significance of Between-Family Trio Differential Methylation

To determine the biological relevance of our results, we considered 51 CpGs on Chromosome 1 that had been previously identified as differentially methylated in an array analysis of approximately 300 individuals from Caucasian-American, African-American, and Han Chinese-American populations [6]. Annotation and methylation results from all 51 CpGs are available within Supplementary Table 5. Of the 7 sites with reported |PMD|>0.2 (Percent Methylation Difference) between Chinese-Americans and Caucasian-Americans, all had corresponding

|PMD|>0.2 within the microarray data. Additionally, 4 of these were identified as statistically significant DMAs across all six sequencing assays (five short read library types and Oxford Nanopore). Of the three remaining sites, the first (on the TAS1R3 promoter) was significantly hypomethylated in the Chinese family for EMSeq, Nanopore, SPLAT, and TrueMethyl, the second (on the PM20D1 promoter) had insufficient read coverage for TruSeq but was a DMA for the remaining assays, and the third (located on the C1orf100 promoter) was identified as a DMA for only SPLAT although estimated PMD values were greater than 0.1 for all assays. Notably, these sites were identified as methylation quantitative trait loci (meQTL) in the original analysis. In addition to TAS1R3, which is a sweetness taste receptor that is known to vary phenotypically between the Asian and Caucasian populations [7], there was strong concordance for 6 CpGs on the PM20D1 promoter, a gene associated with obesity and Alzheimer's disease with demonstrated population based variation [8, 9].

We additionally reviewed the collection of 29,802 sites on Chromosome 1 that were identified as differentially methylated for four or more of the six sequencing assays. Following annotation with HOMER [10], analysis with DAVID [11] identified a subset of 133 genes associated with hypertension (Benjamini Hochberg adjusted *p*-value = 5.0E-13), 54 genes associated with osteoporosis (*p* = 5.0E-13), and 18 genes associated with atopic dermatitis (*p* =1.0E-5) according to the GAD database [12]. Only 1204 (4.0%) of these sites were included on the Infinium MethylEPIC array, and while annotation for these sites included 53 of the hypertension-associated genes (*p*=3.3E-4) and 9 of those associated with atopic dermatitis (*p*=0.03), only 17 of the genes identified with osteoporosis were included and this was an insufficient number to result in a significant association.

EMSeq Input Titration

In order to investigate the impact of input DNA on detection and characterization of CpG methylation, we generated EM-Seq libraries using 10ng, 50ng, and 100ng aliquots of input DNA for each replicate for each member of the Chinese Han Trio in this study (HG005-7). We then randomly subsampled each run *in silico* to a random set of 1M, 5M, 10M, 25M, 50M, and 100M paired end 150bp reads per input. At the lowest read input, the less complex 10ng library covered CpGs greater than 50ng and 100ng libraries, though beyond 25M paired end reads the more complex (50/100ng) libraries surpassed the 10ng library in mean CpG coverage (**Additional file 1: Figure S13a**). All three library types exhibited similar distributions of CpG coverage across read titrations, reflecting fringe technical noise contributing to mean depth differences at low inputs that were evened out with more input. This was further validated by looking at the intersection of CpGs covered by each input type at each read filtration titer, where by 10M paired end reads the majority of sites were shared by all libraries, and notably the lowest input consistently covered the fewest unique CpGs (**Additional file 1: Figure S13c**).

Methyl EPIC Capture Correlations

We compared the whole epigenome libraries to sequencing replicates of Illumina Methyl Capture EPIC, a

reduced representation bisulfite approach interrogating roughly 3.3 million CpGs with a preference for CpG islands and promoter regions. Results shown for HG002 are representative of all seven genomes. Methylation percentage of CpGs within replicates of Capture EPIC were compared to shared sites among whole methylome assays as well as Nanopore sequencing, with good Pearson correlation for all comparisons (average r=0.85). Capture EPIC tended to overestimate fully methylated sites that were estimated to be closer to 50-90% in other assays (**Additional file 1: Figure S14s**).

Using 20X downsampled methylation data, the shared CpG coverage on Chromosome 1 in Capture EPIC sites was highly consistent with overall methylome coverage (**Figure 2**). Nanopore missed the fewest sites covered by EPIC (n=5,179), while TruSeq missed the most (n=21,712).

Supplemental References

1. Krueger F, A. S. Bismark: a flexible aligner and methylation caller for Bisulfite-Seq applications. *Bioinformatics* **27,** 1571–2 (2011).
2. Cheng, H. & Xu, Y. BitMapperBS: a fast and accurate read aligner for whole-genome bisulfite sequencing. *bioRxiv.* eprint: https://www.biorxiv.org/content/early/2018/10/14/442798.full.pdf. https://www.biorxiv.org/content/early/2018/10/14/442798 (2018).
3. Guo, W. *et al.* BS-Seeker2: a versatile aligning pipeline for bisulfite sequencing data. *BMC genomics* **14,** 1–8 (2013).
4. (https://github.com/brentp/bwa-meth).
5. Merkel A Fernández-Callejo M, C. E. M.-S. S. S. R. G. I. H. S. gemBS: high throughput processing for DNA methylation data from bisulfite sequencing. *Bioinformatics* **35,** 737–742 (2019).
6. Heyn, H. *et al.* DNA methylation contributes to natural human variation. *Genome Res.* **23,** 1363–1372 (2013).
7. Fushan, A. A., Simons, C. T., Slack, J. P., Manichaikul, A. & Drayna, D. Allelic polymorphism within the TAS1R3 promoter is associated with human taste sensitivity to sucrose. *Curr. Biol.* **19,** 1288–1293 (2009).
8. Sanchez-Mut, J. V. *et al.* PM20D1 is a quantitative trait locus associated with Alzheimer's disease. *Nat. Med.* **24,** 598–603 (May 2018).
9. Benson, K. K. *et al.* Natural human genetic variation determines basal and inducible expression of PM20D1, an obesity-associated gene. *Proceedings of the National Academy of Sciences* **116,** 23232–23242. ISSN: 0027-8424. eprint: https://www.pnas.org/content/116/46/23232.full.pdf. https://www.pnas.org/content/116/46/23232 (2019).
10. Heinz, S. *et al.* Simple combinations of lineage-determining transcription factors prime cis-regulatory elements required for macrophage and B cell identities. *Mol. Cell* **38,** 576–589 (2010).
11. Huang, d. a. W., Sherman, B. T. & Lempicki, R. A. Bioinformatics enrichment tools: paths toward the comprehensive functional analysis of large gene lists. *Nucleic Acids Res.* **37,** 1–13 (2009).
12. Becker, K. G., Barnes, K. C., Bright, T. J. & Wang, S. A. The genetic association database. *Nat. Genet.* **36,** 431–432 (2004).

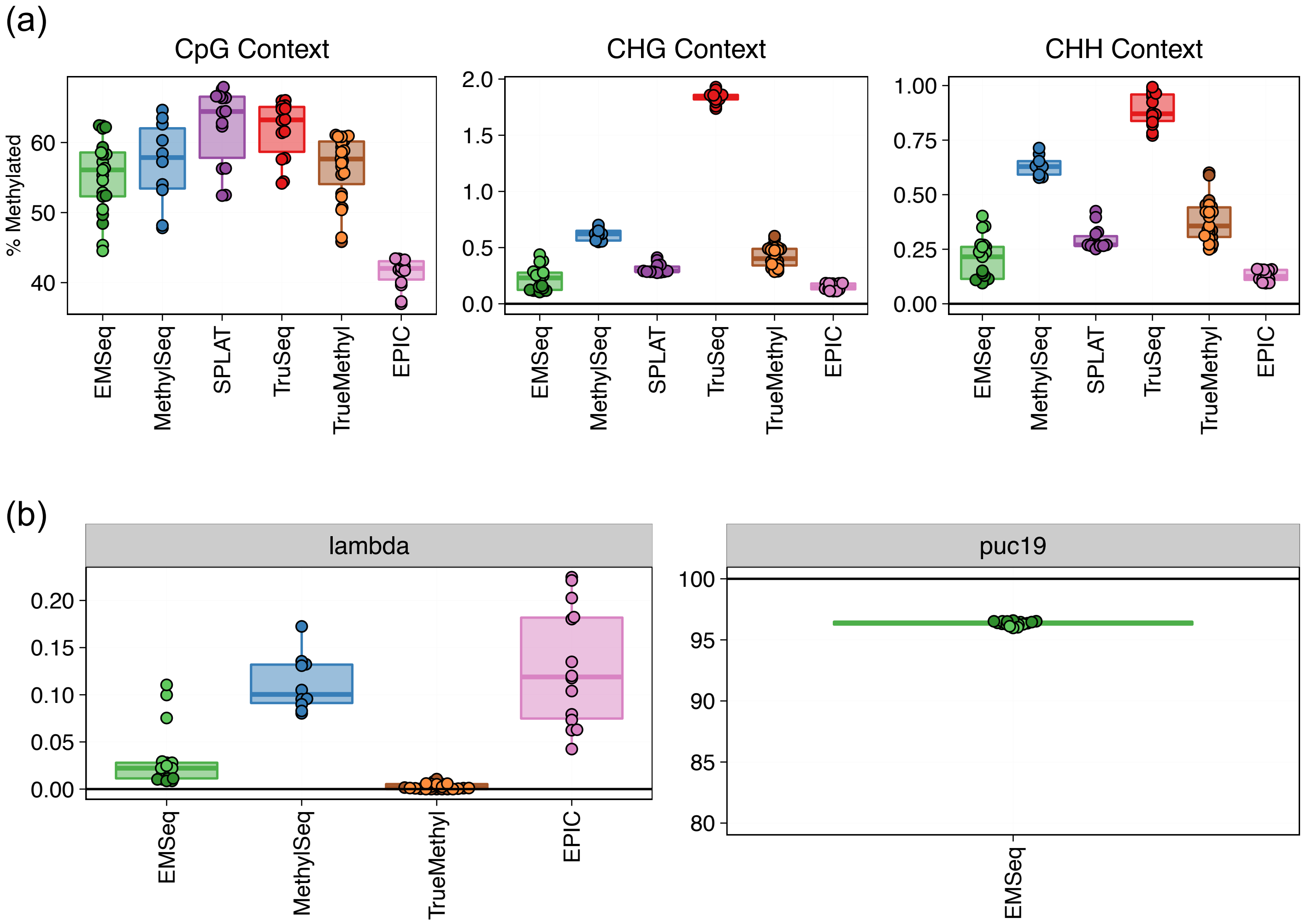

Figure S1: Measurement of sequencing control samples (a) Estimated methylation percentage in CpG, CHG, and CHH contexts per assay. Efficient conversion results in near-zero converted cytosines in CHG and CHH contexts. (b) Estimated methylation percentage in unmethylated controls, showing only assays that had these controls spiked in as a part of their library preparation.

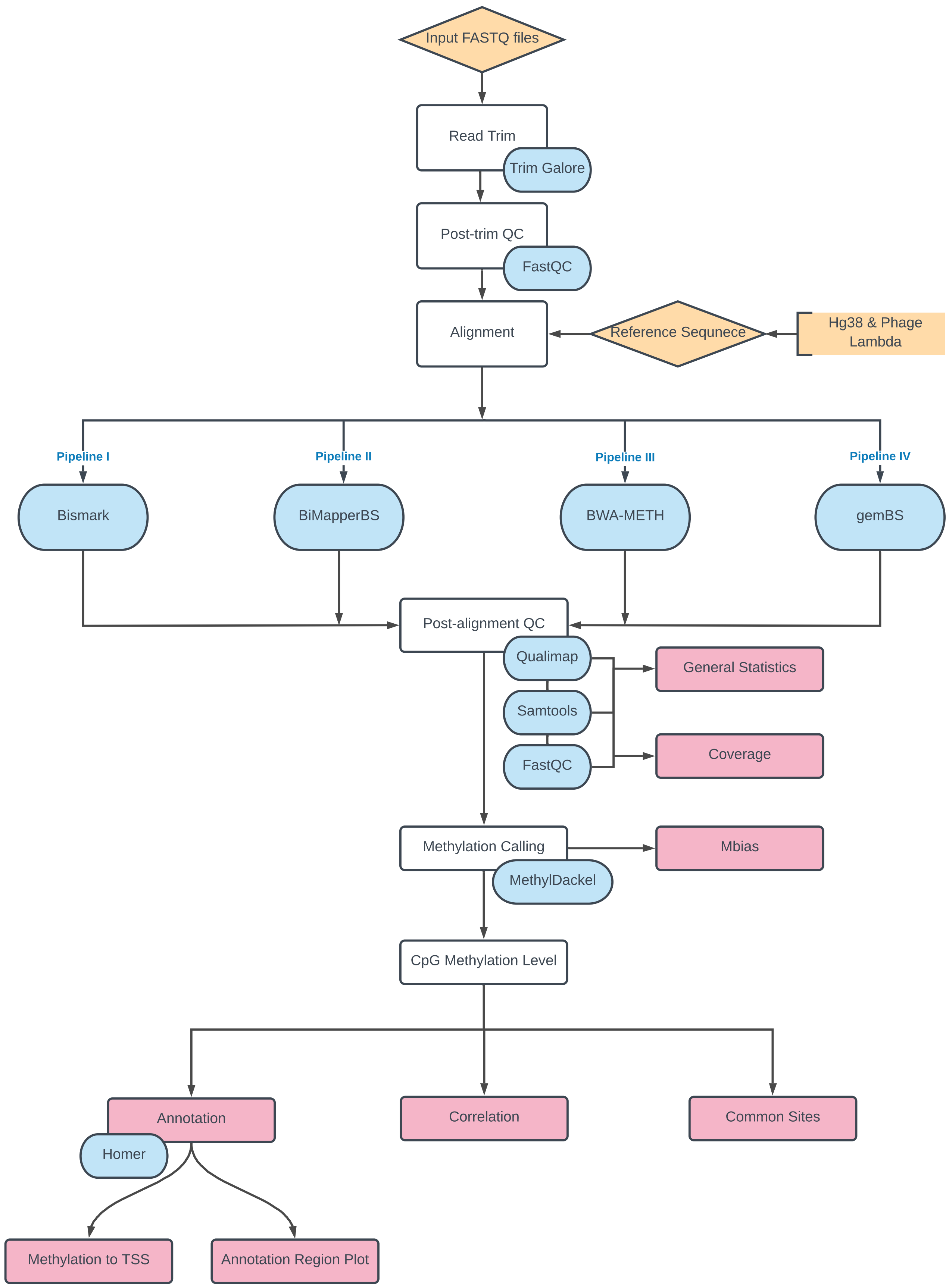

Figure S2: Flowchart showing recommended steps for read quality control, reference-based read alignment, and methylation extraction, for each methylation package analyzed.

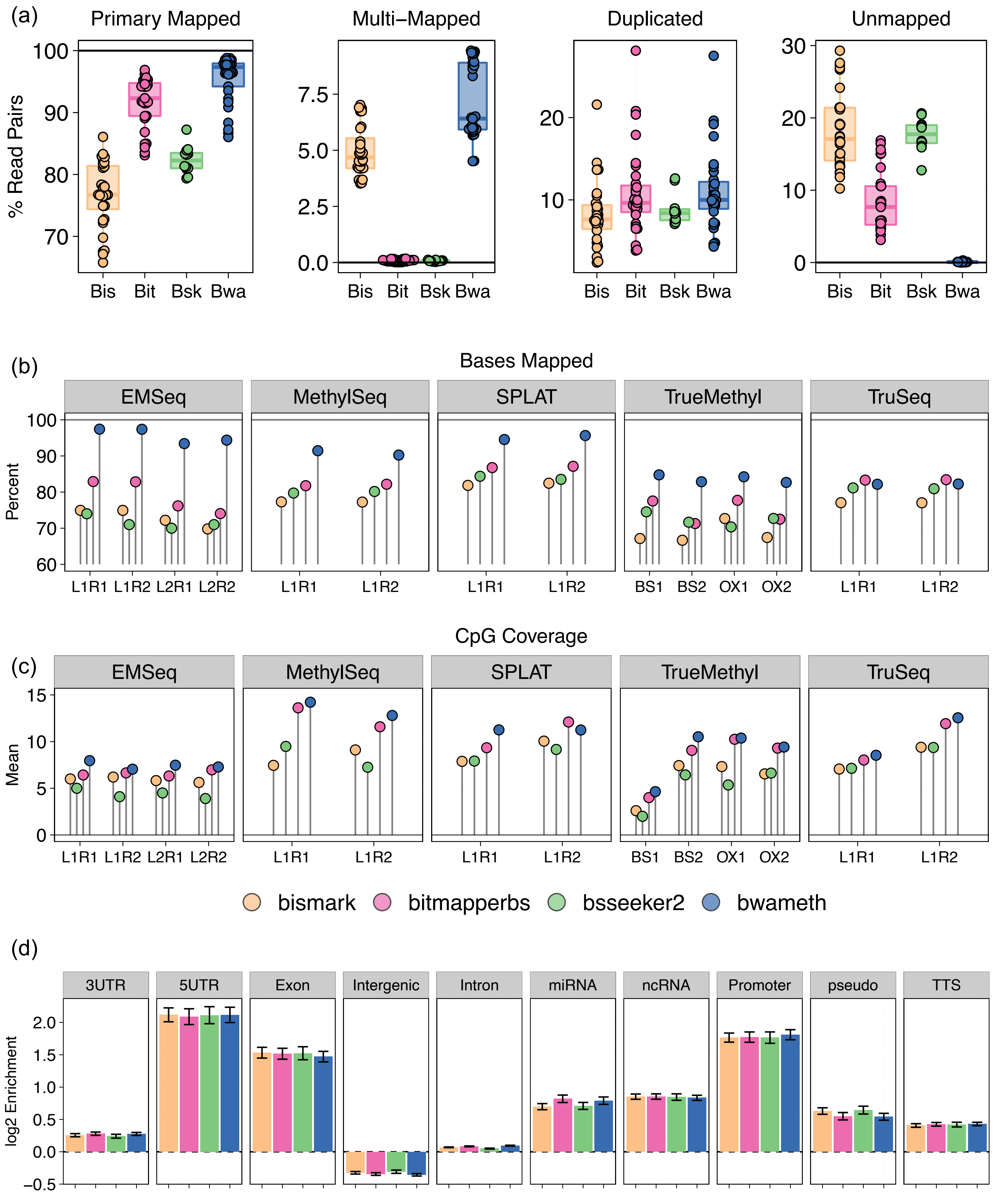

Figure S3: Comparison of outputs for each methylation detection pipeline. All figures show analysis of all HG002 samples for each short read epigenomic assay. (a) Distribution of reference-based read alignment outcomes, including primary mapped reads (both mates mapped in correct orientation within a certain distance), multi-mapped reads (read pairs containing secondary or supplementary alignments), reads marked as PCR or optical duplicates, and unmapped reads. Ambiguous and duplicate reads can be a subset of properly aligned reads. (b) Mapping efficiency per pipeline as measured by the total percentage of reads aligned to the reference genome. L1 and L2 = Lab 1/2; R1 and R2 = Replicate 1/2; BS1 and BS2 = bisulfite treatment replicates 1/2; OX1 and OX2 = oxidative-bisulfite replicates 1/2. (c) The mean coverage per CpG across the genome per pipeline. (d) The regions of the genomes covered per pipeline, measured as log2 enrichment against a null genomic distribution.

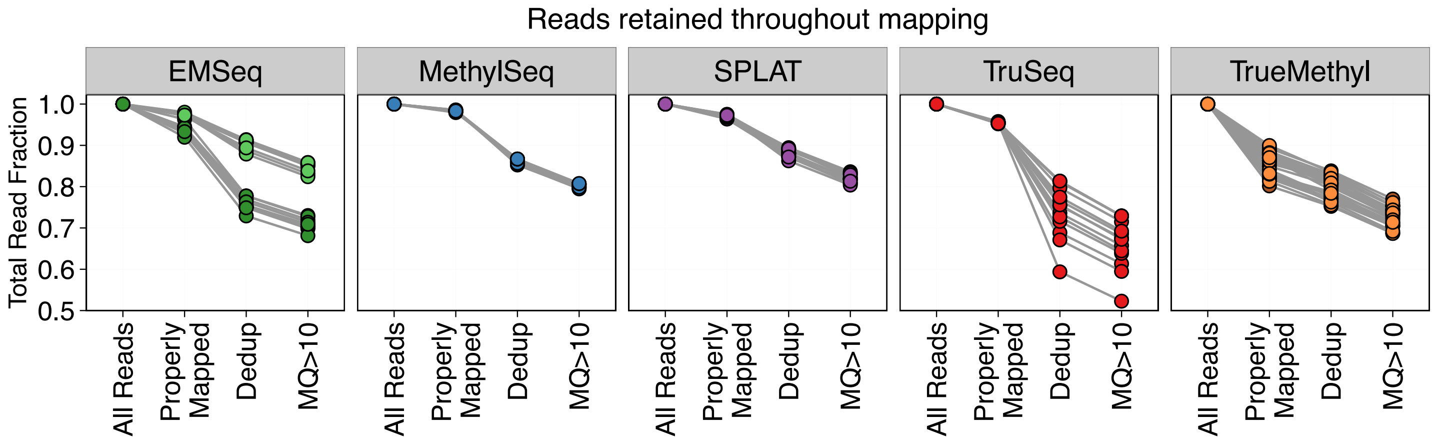

Figure S4: Read retention rate. The fraction of total reads that are retained after each step of the epigenome alignment process is shown per assay. Properly mapped = both mates of a pair were mapped in the correct orientation within a 1kb distance. Dedup = removing reads that are marked as duplicates. MQ = Mapping Quality.

a)

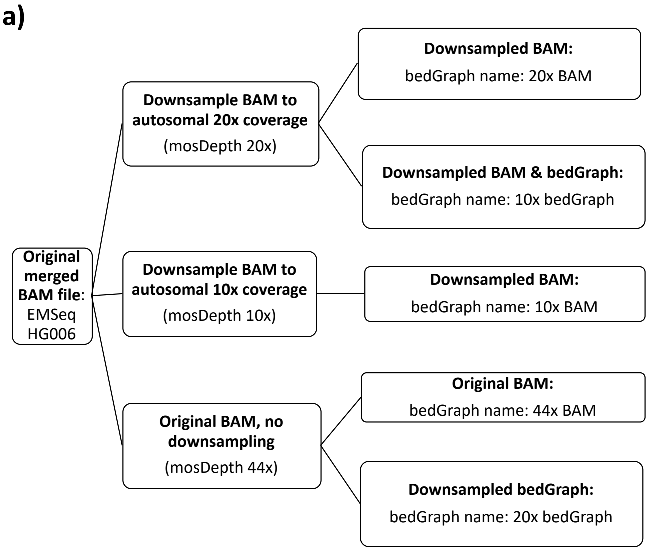

b)

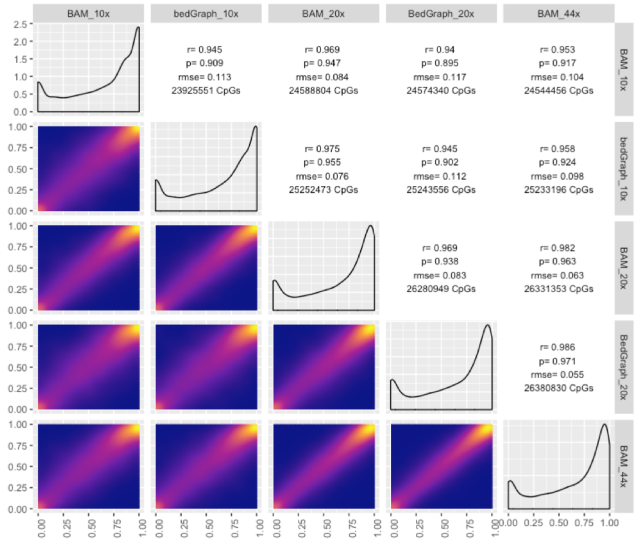

c)

bedGraph name:	10x BAM	10x bedGraph	20x BAM	20x bedGraph	44x BAM
Type of down-sampling	BAM	BAM (20x) -> bedGraph (10x)	BAM	bedGraph	none
Final target coverage	10x	10x	20x	20x	44x
**Beta-value statistics**					
Min.	0.00	0.00	0.00	0.00	0.00
1st Qu.	0.36	0.36	0.35	0.42	0.43
Median	0.77	0.77	0.76	0.79	0.79
Mean	0.64	0.64	0.63	0.66	0.66
3rd Qu.	1.00	1.00	0.94	0.94	0.93
Max.	1.00	1.00	1.00	1.00	1.00
**Coverage statistics**					
Min.	1	1	1	1	1
1st Qu.	7	7	14	16	33
Median	9	10	18	20	39
Mean	9.0	10.0	17.9	20.4	38.4
3rd Qu.	11	12	21	24	44
Max.	1930	1130	1932	2946	5478
99%	18	19	31	35	60
95%	15	16	27	30	53

d)

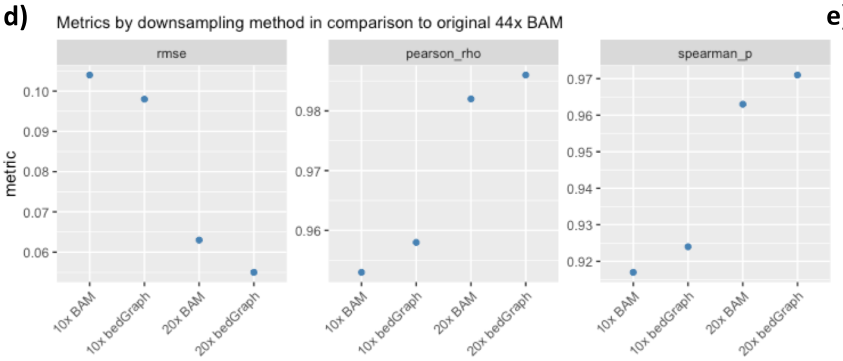

e)

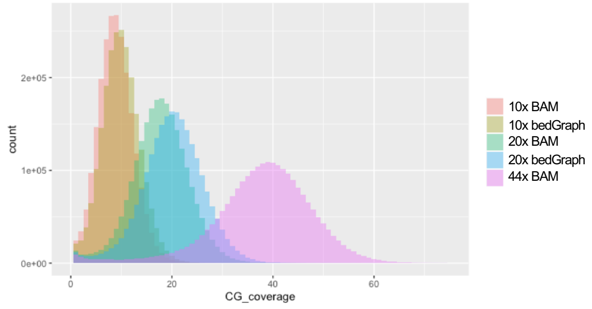

Figure S5: Downsampling evaluation for EMSeq / HG006. (a) Outline of the downsampling procedure and naming scheme of the downsampled libraries. (b) Pairwise correlation matrix of methylation values for the EMSeq HG006 library from Lab 1. Scatter plots of the methylation values are shown in the lower left. Histograms of the methylation values per library are shown across the diagonal. Pairwise Pearson (rho) and Spearman (p) correlation coefficients, root mean square error (RMSE), and the number of CpG dinucleotides with >= 5x coverage in both libraries are shown in the upper right. (c) Statistics over the methylation percentage distributions and observed read coverage of CpG sites in the various bedGraph files. (d) RMSE, Pairwise Pearson (p) and Spearman (rho) correlations between downsampled BAM and bedGraph files in comparison to the original 44x average coverage BAM file. (e) Histograms of the CG dinucelotide read coverage of each bedGraph file prior (44x BAM) to and after downsampling the BAM or bedGraph.

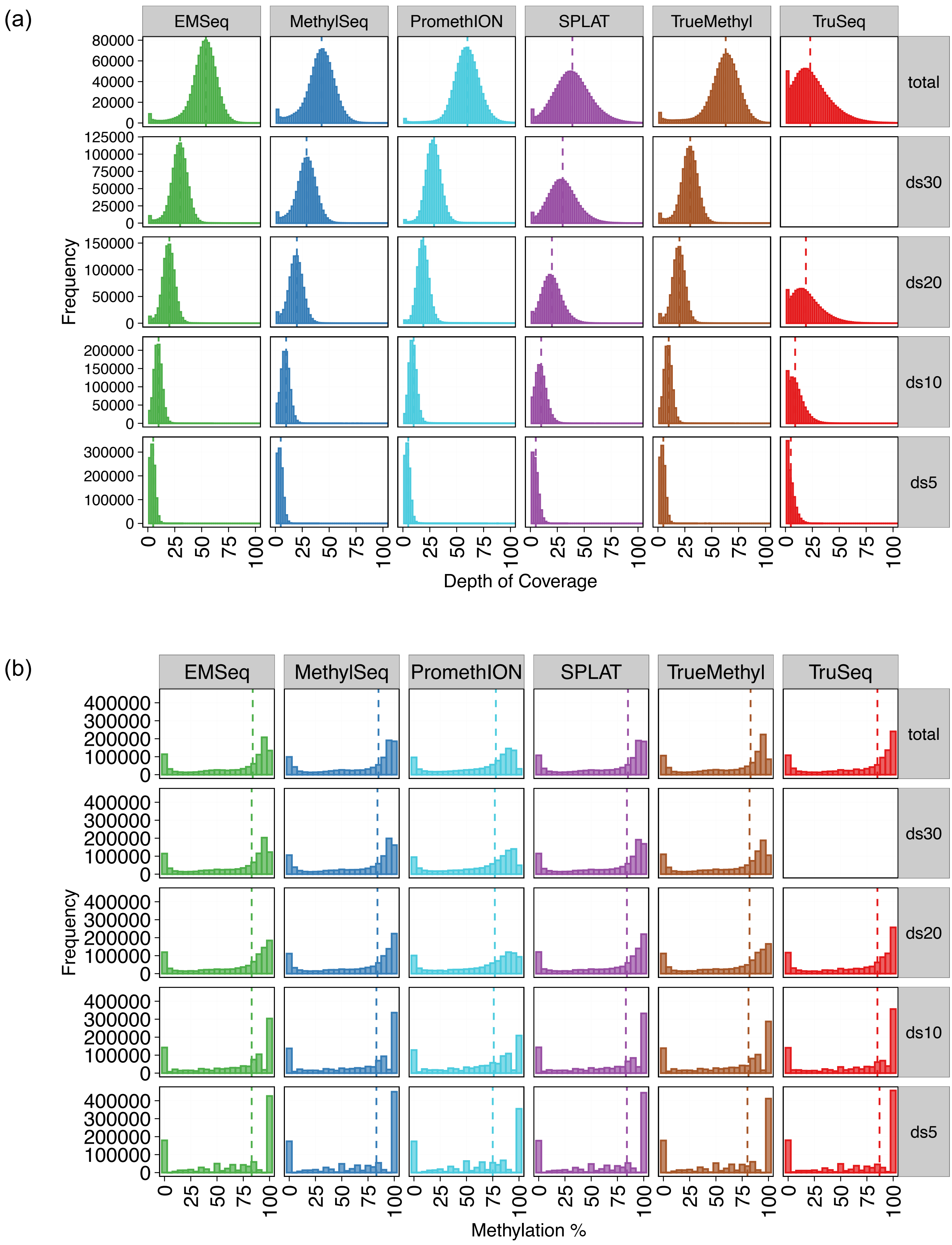

Figure S6: CpG coverage and methylation percentage distributions for complete and downsampled libraries per assay. All values are shown for replicates of HG003. ds = downsample, indicating the mean CpG coverage samples were normalized to. Vertical dotted lines indicate median coverage/methylation percentage. TruSeq could not be downsampled at the 30x level because the total mean depth for TruSeq libraries equaled roughly 30x.

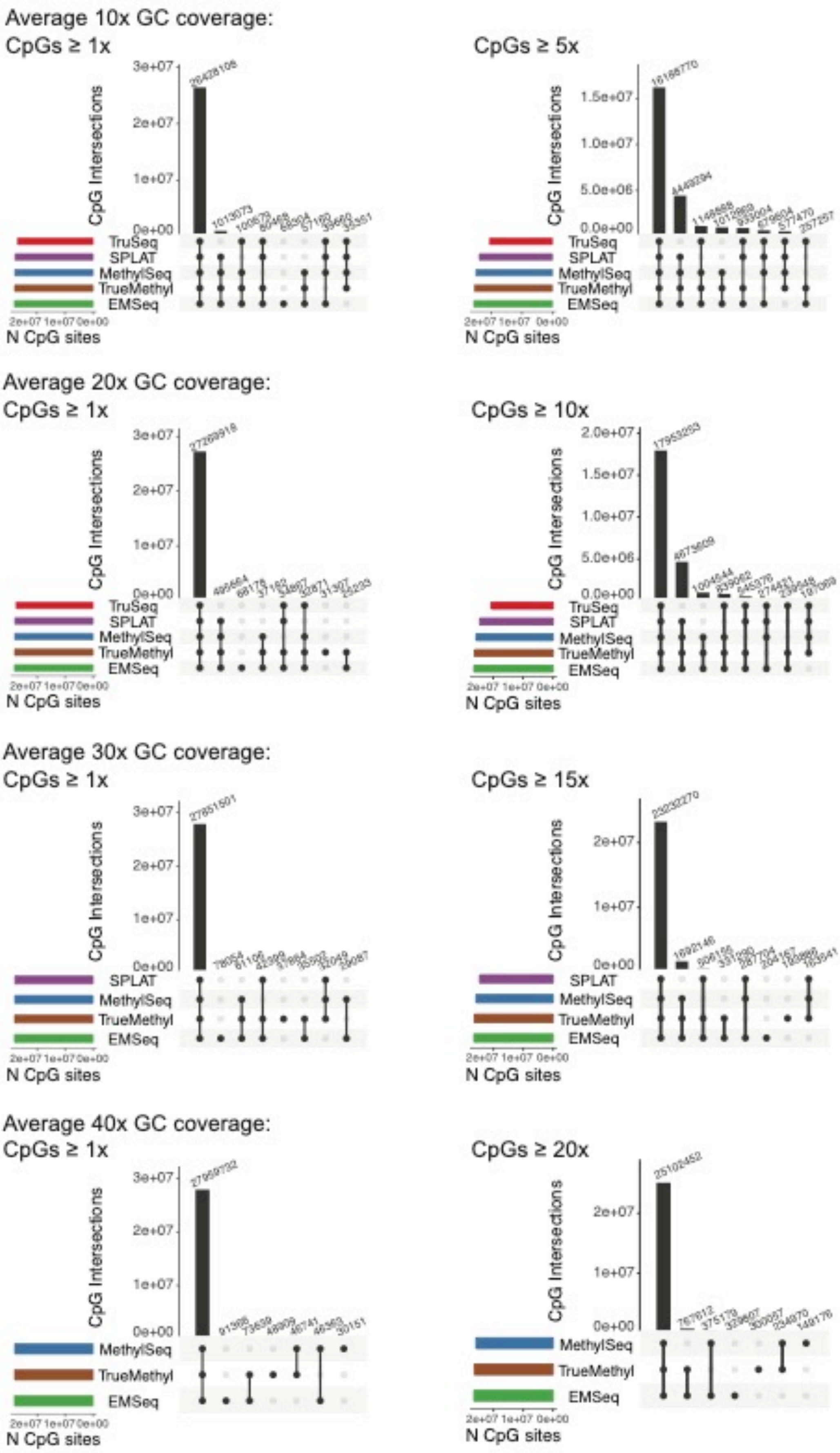

Figure S7: UpSet plots showing shared coverage of CpGs across assays across downsampling schema, with a minimum of 1x cov per CpG on the left and a minimum of 50% of the downsampling scheme on the right (e.g. minimum of 5x coveage for 10x downsampled data).

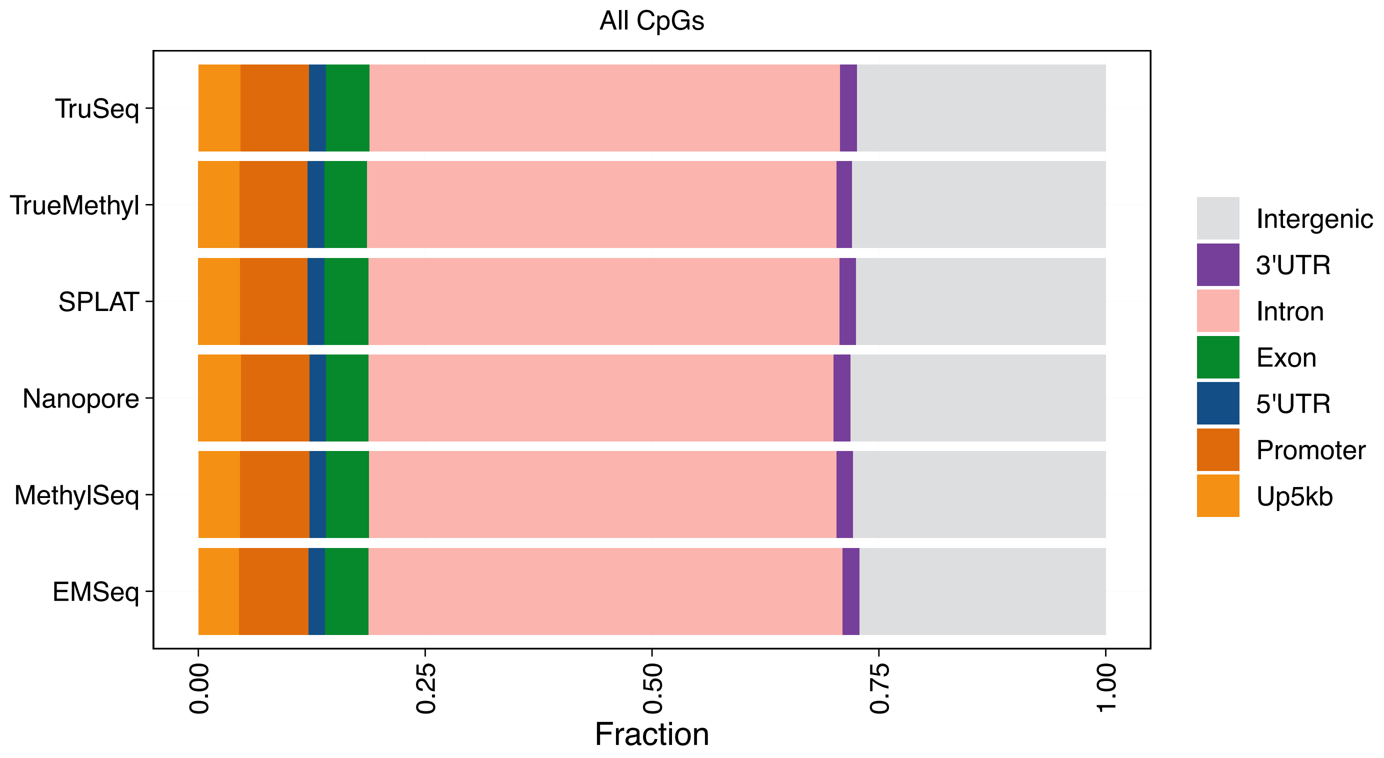

Figure S8: Annotating CpGs covered by each assay using normalized mean 20x coverage data, showing the consistency of coverage genome-wide. Up5kb = 5kb upstream of promoter regions. Promoter = 1kb upstream of transcript start sites.

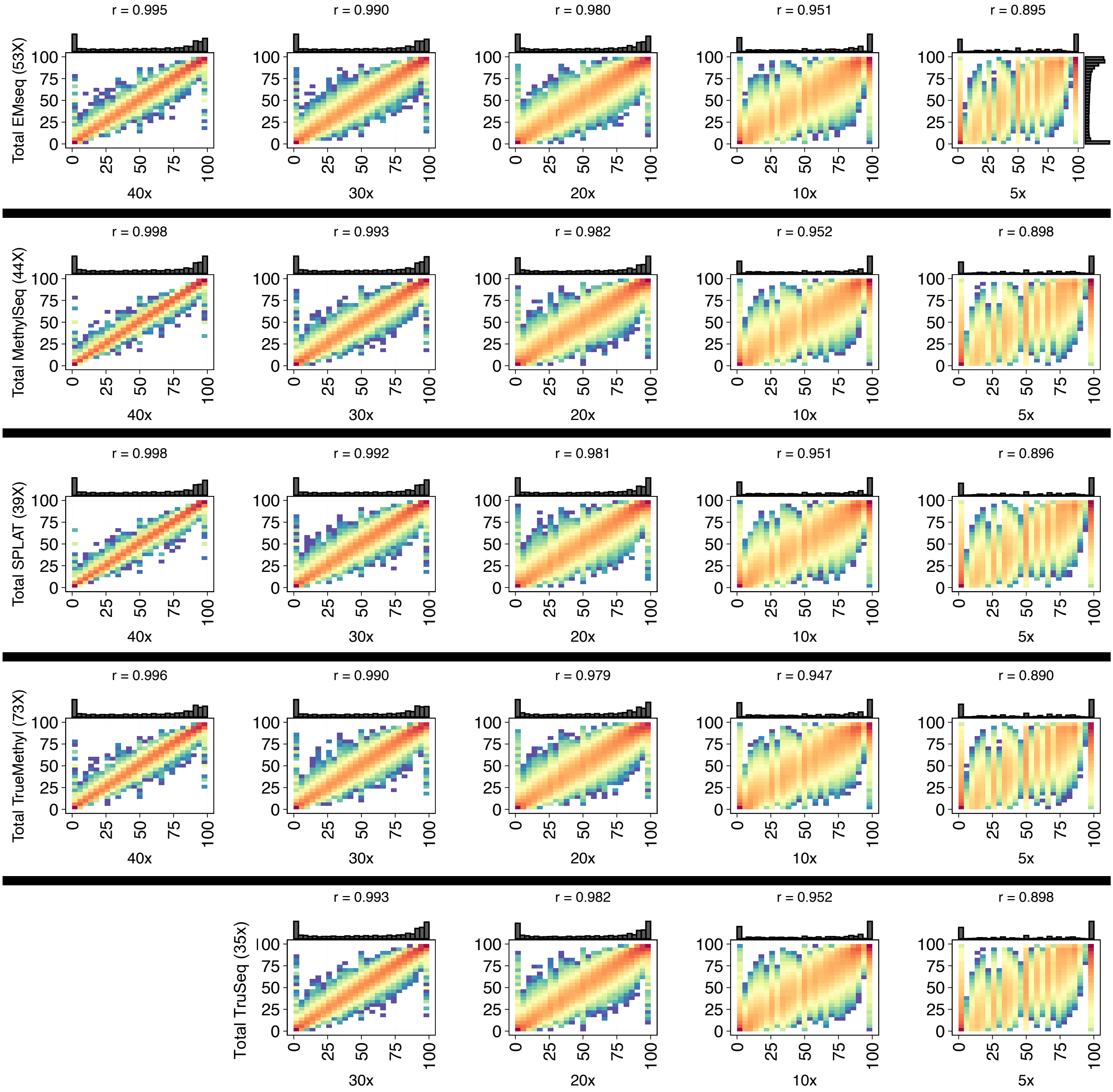

Figure S9: Pearson correlations of methylation percentage estimation within each assay, comparing the total data (y- axes) against their respective downsampled schema (x-axes), for combined replicates of HG002 libraries. Pearson values are shown above each comparison, as well as marginal histograms showing methylation percentage distributions. For TruSeq, the total data returned a mean coverage of 35X, meaning that a comparison to 40X downsampling was not possible.

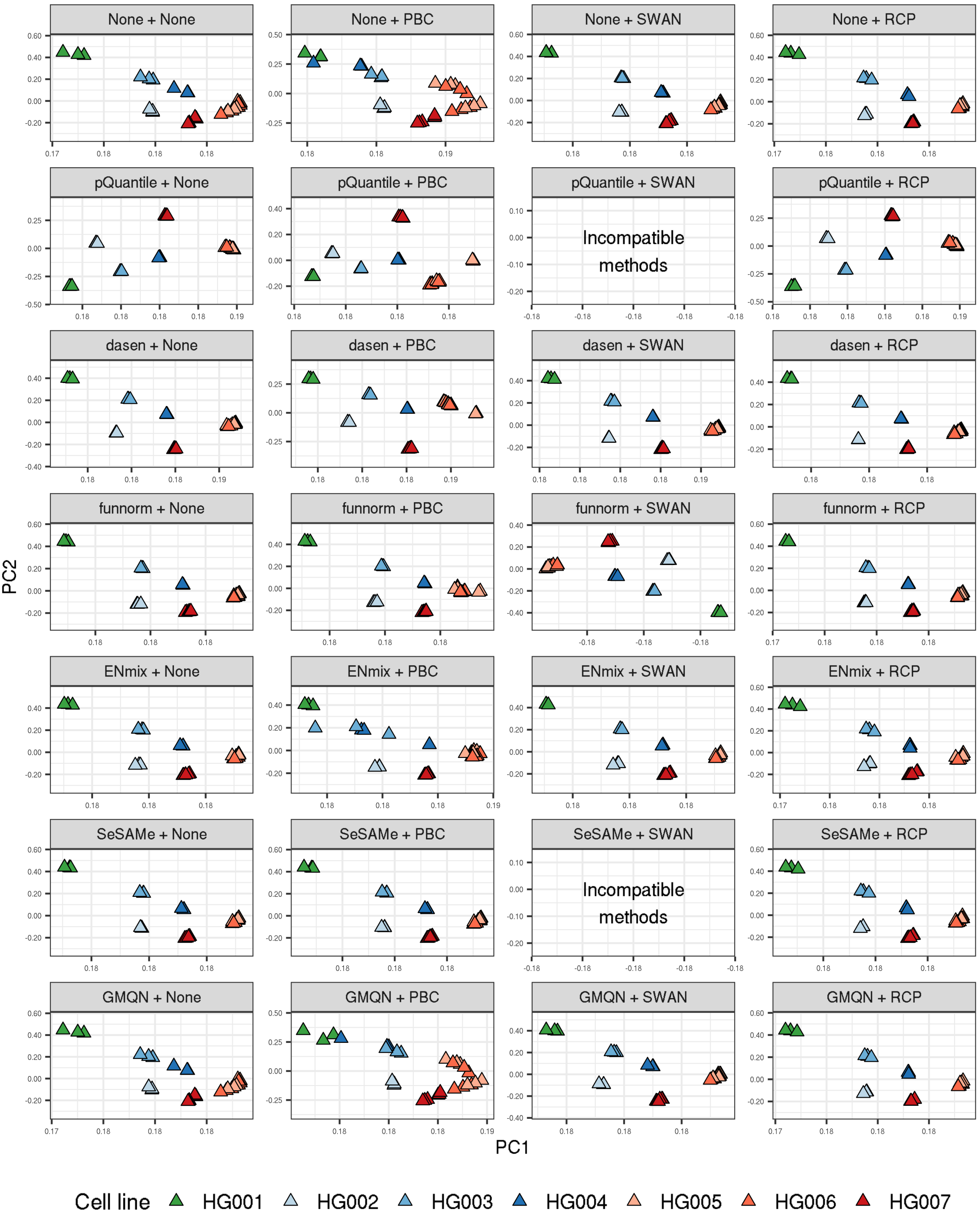

Figure S10: First two principal components (PCs) calculated from 678,597 CpG sites with complete information in all normalized microarray datasets, by normalization pipeline.

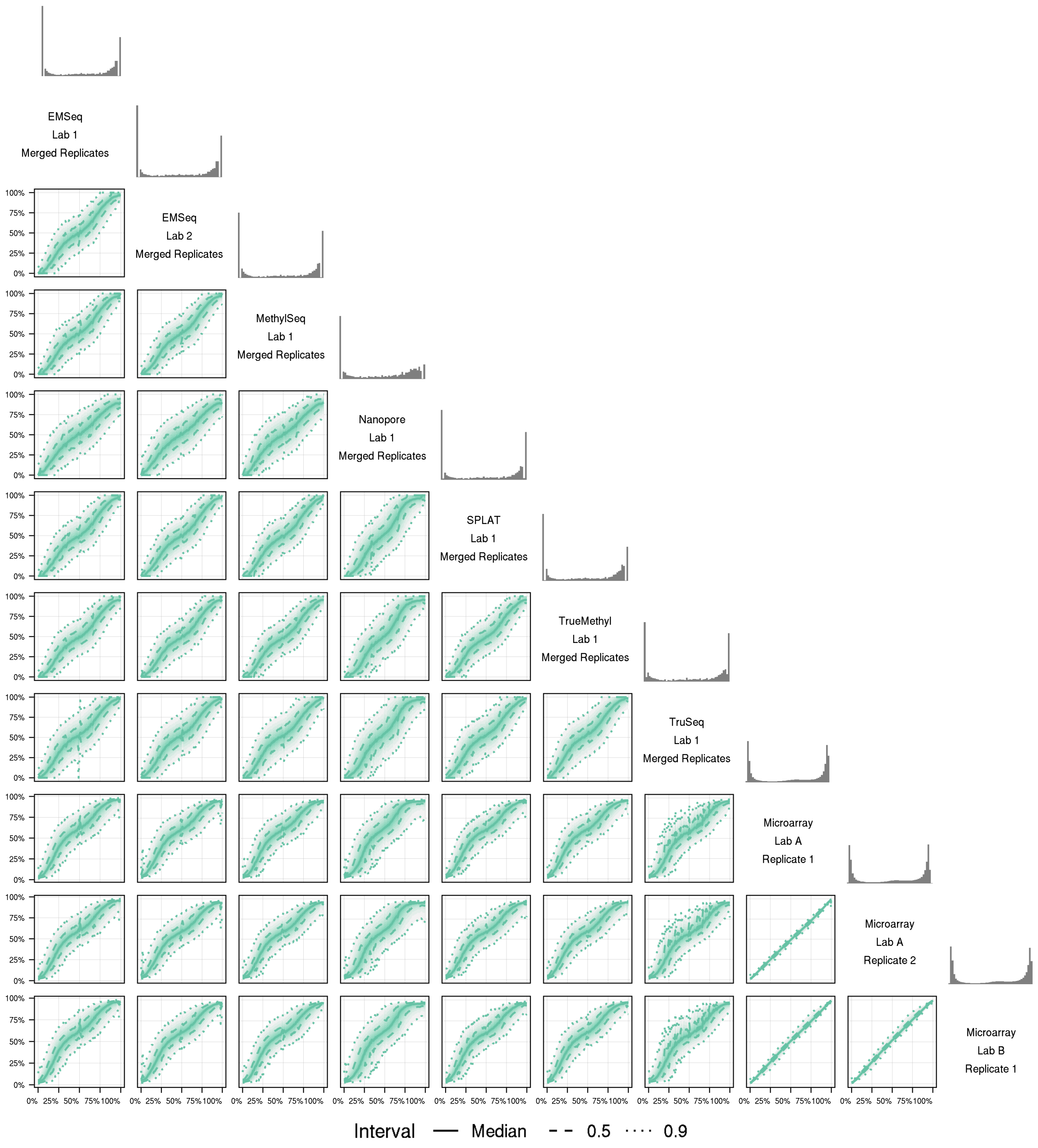

Figure S11: Distribution of beta values across HG002 samples at 841,833 CpG sites with complete information in all assays. Beta values for the assay on the x axis were binned (binwidth=0.01) to calculate beta value deciles for the assay on the y axis, indicated by the color transparency. 90% of the y-axis values fall between the outermost dotted lines for each bin along the x-axis. Marginal histograms for each assay are shown above the assay label.

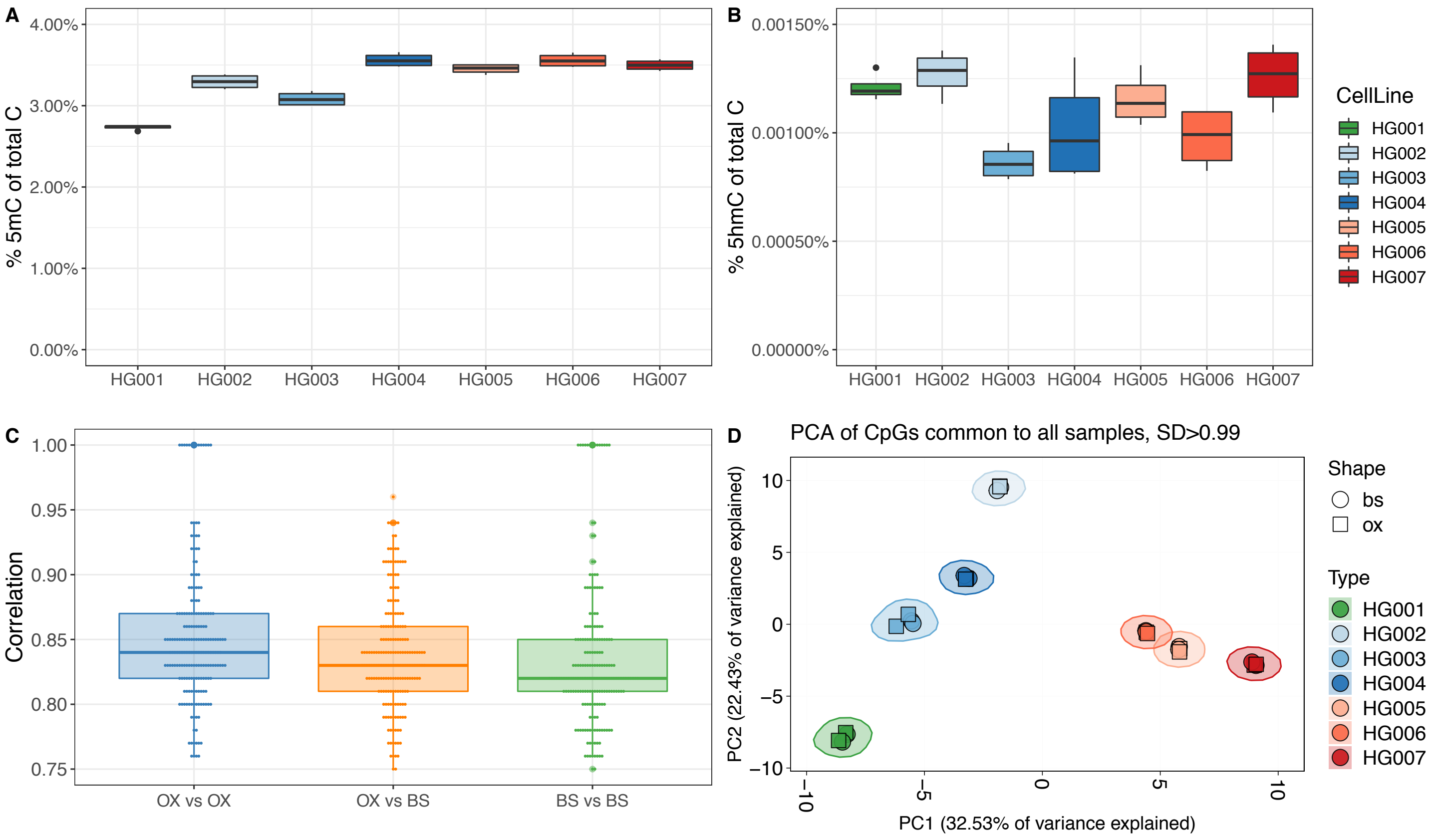

Figure S12: Capture of 5mC and 5hmC from TrueMethyl replicates, including bisulfite-only (bs) and oxidative bisulfite (ox). (a) Percent of inferred 5mC among all cytosines in the genome. (b) Percent of inferred 5hmC among all cytosines in the genome. (c) Pearson correlation of replicates across genomes between oxidative and bisulfite replicates. (d) Unsupervised clustering of samples, including OX and BS samples.

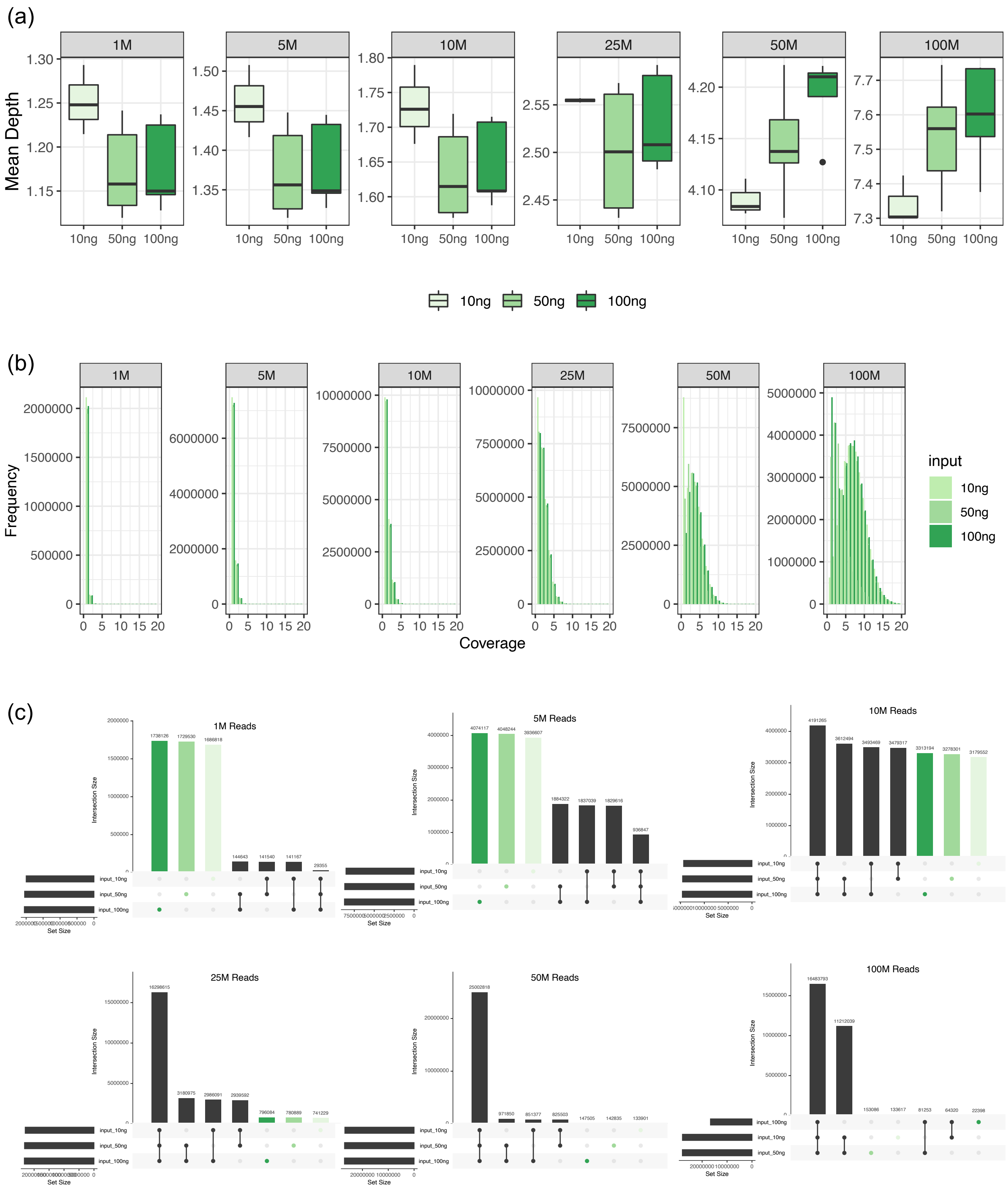

Figure S13: EM-Seq read titration experiment. Replicates generated using 10ng, 50ng, and 100ng of input DNA for HG005, HG006, and HG007 were randomly downsampled to 1M, 5M, 10M, 25M, 50M, and 100M paired end 150bp input reads. (a) Distribution of mean depth of CpGs covered for each input amount. (b) Read coverage distributions per input type per downsampled read amount. (c) UpSet plots showing the intersections of CpGs shared by each downsampling scheme, as well as uniquely covered CpGs.

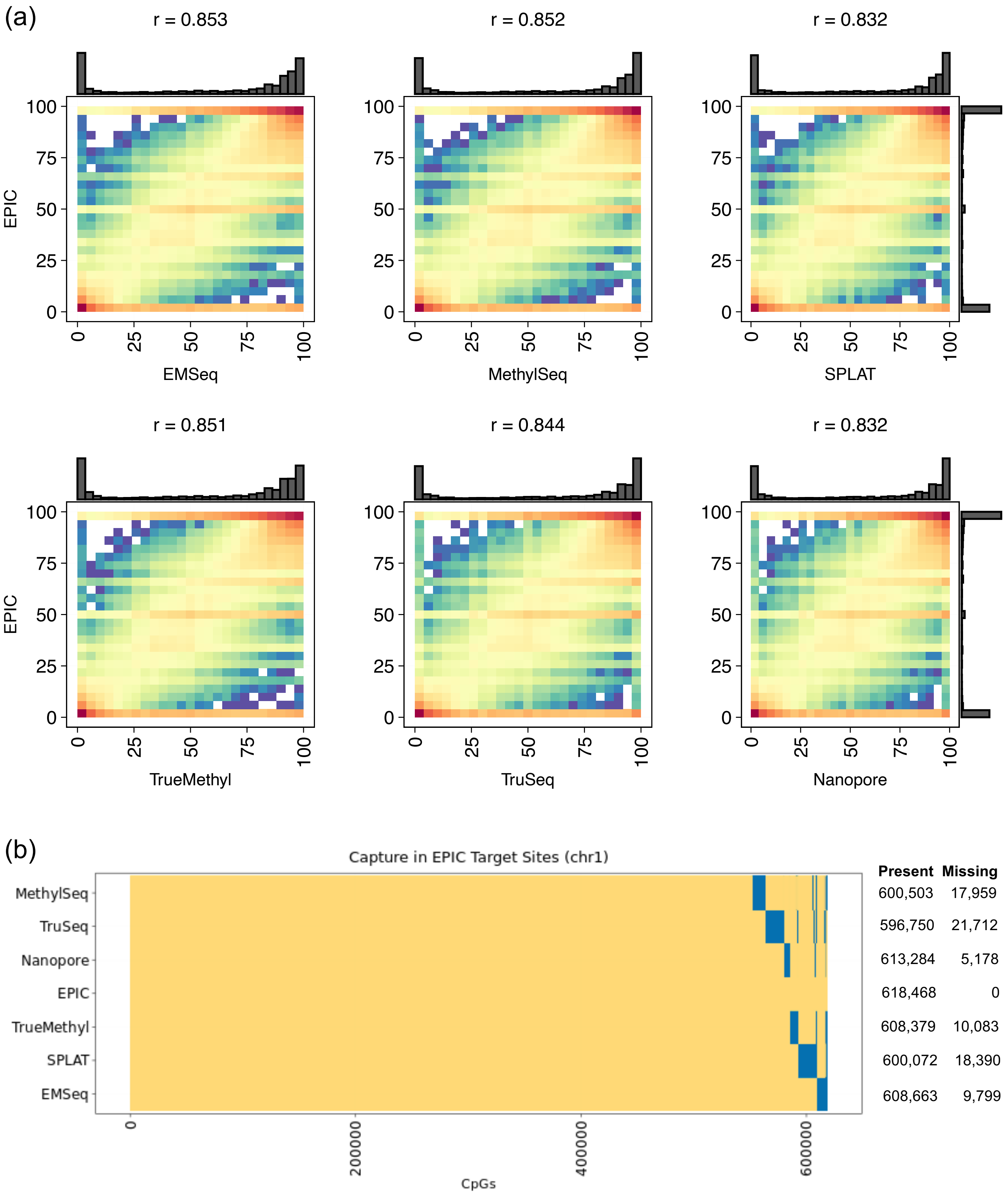

Figure S14: (a) Pearson correlation of percent methylation estimates of Methyl Seq EPIC Capture versus each whole methylome library. All values are shown for Chromosome 1 of HG002 replicates. (b) Distribution of CpGs covered (in yellow) or missed (in blue) by each assay on Chromosome 1. Total values are shown per assay in the table on the right.
